# Method validation and analysis of halogenated natural products (HNPs) in seafood samples

**DOI:** 10.1007/s00216-025-06141-2

**Published:** 2025-10-08

**Authors:** Marco Krämer, Walter Vetter, Oliver Kappenstein, Astrid Spielmeyer

**Affiliations:** 1 Department Reference Centre for Food and Feed Analysis, German Federal Institute for Risk Assessment, National Reference Laboratory for the Monitoring of Marine Biotoxins, Max-Dohrn-Str. 8-10, 10589 Berlin, Germany; 2 Institute of Food Chemistry (170B), University of Hohenheim, Garbenstraße 28, 70599 Stuttgart, Germany

**Keywords:** Halogenated natural product, Persistent organic pollutant, Seafood, Freshwater fish, Crustaceans, Molluscs

## Abstract

Halogenated natural products (HNPs) are a diverse group of organohalogen compounds, predominantly found in the marine environment. Selected HNPs show structural similarity to persistent organic pollutants (POPs), but data on their occurrence is limited. An analytical method for the detection of 17 commonly reported HNPs, as well as selected POPs, using gas chromatography with electron-capture negative ionisation mass spectrometry (GC/ECNI-MS) was established and validated for salmon and blue mussel. The validation confirmed the applicability of the method with recovery rates and inter-day coefficients of variations of 52–101% and 3.7–16%, respectively. The subsequent application of the method to 17 samples of frequently consumed seafood products from the German retail market demonstrated its suitability for a wide variety of matrices. HNPs were detected in all analysed samples, with contents ranging from <0.05 to up to 7000 ng/g lipid weight (lw). Highest median values were found for mixed-halogenated compound 1 (MHC-1), 2,4,6-tribromoanisole (2,4,6-TBA), and the heptachlorinated bipyrrole Q1 (19, 6.0, and 3.4 ng/g lw, respectively). In 14 out of 17 analysed samples, the sum of HNPs was in the same concentration range or exceeded the sum of the investigated POPs, indicating the relevant exposure of consumers of seafood products in Germany to HNPs and emphasising the relevance of HNPs as organohalogen compounds in seafood samples.

**Supplementary Information:**

The online version contains supplementary material available at 10.1007/s00216-025-06141-2.

## Introduction

Halogenated natural products (HNPs) represent a diverse array of organohalogen compounds, with the majority containing one or more chlorine or bromine substituents. More than 8000 HNPs have been discovered, and the number continues to grow [1]. A small group of HNPs shows structural similarity with persistent organic pollutants (POPs), such as polybrominated diphenyl ethers (PBDEs) or polychlorinated biphenyls (PCBs), raising the question of potential bioaccumulation of and consumer exposure to HNPs [2]. In certain regions, HNP contents were found to be in the same order of magnitude as POPs, which gave reasons for concerns about possible adverse effects on human health and the environment [3]. Thus, the Arctic Monitoring and Assessment Programme (AMAP) classified HNPs as Chemicals of Emerging Arctic Concern [4]. Most of these POP-like HNPs were discovered in the marine environment, and the most relevant natural producers are algae, sponges, fungi, and bacteria [1]. Their natural origin has been demonstrated, amongst others, by isotope composition and detection in samples from before the advent of the organohalogen industry [5–7].

**Fig. 1:**
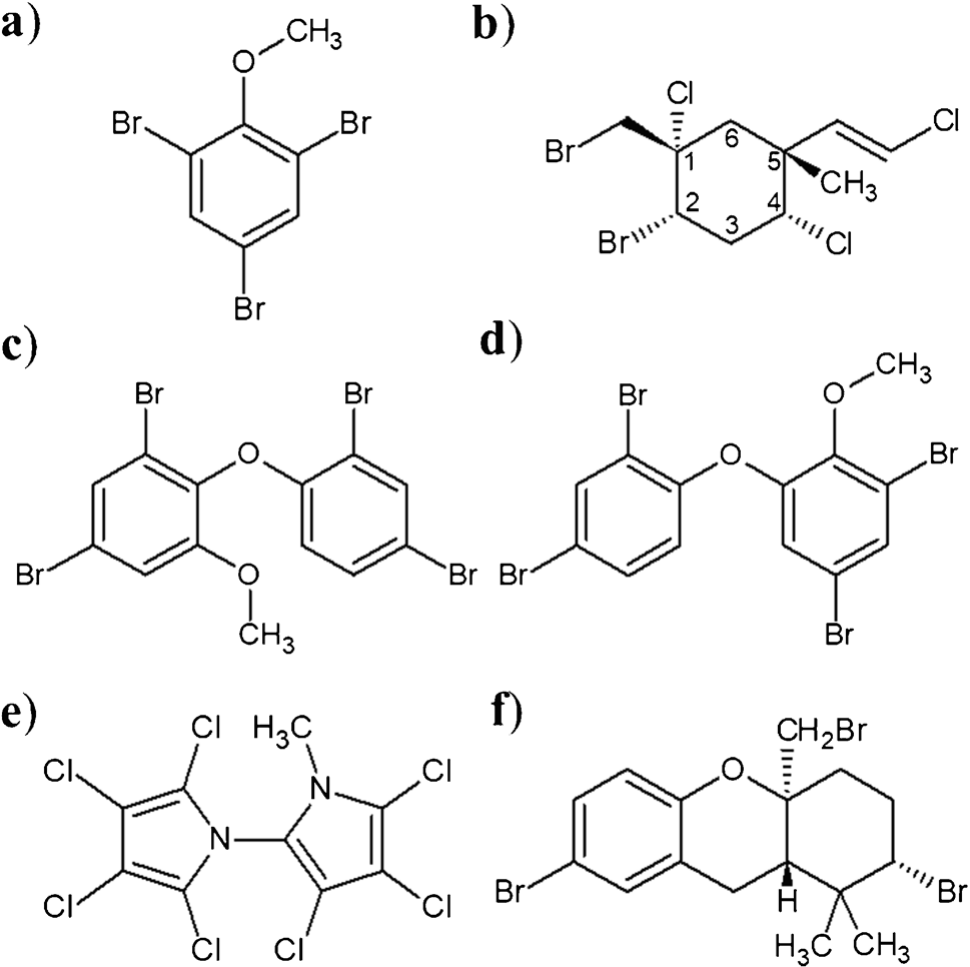
Structures of selected HNPs with **a** 2,4,6-tribromanisol (2,4,6-TBA), **b** (1*S*,2*S*,4*R*,5*R*,1′*E*)−2-bromo-1-bromomethyl-1,4-dichloro-5-(2′-chloroethenyl)−5-methylcyclohexane (mixed-halogenated compound 1, MHC-1), **c** 6-methoxy-2,2′,4,4′-tetrabromodiphenyl ether (6-MeO-BDE 47 or BC-3), **d** 2′-methoxy-2,3′,4,5′-tetrabromodiphenyl ether (2´-MeO-BDE 68 or BC-2), **e** 2,3,3′,4,4′,5,5′-heptachloro-1′-methyl-1,2′-bipyrrole (Q1), and **f** (2*S*,4a*S*,9a*S*)−2,7-dibromo-4a-bromomethyl-1,1-dimethyl-2,3,4,4a,9,9a-hexahydro-1*H*-xanthene (TriBHD). Note that the stereocenter at C1 of MHC-1 was erroneously referred to 1*R* instead of 1*S* in some publications

HNPs have been described in numerous higher trophic organisms, among others in fish [8–10], bivalves [11–13], and marine mammals [14–16]. Substances frequently reported include 2,4,6-tribromanisol (2,4,6-TBA), the mixed-halogenated compound 1 (MHC-1), brominated phenoxyanisoles (MeO-BDE) like 6-MeO-BDE 47 (BC-3) and 2′-MeO-BDE 68 (BC-2), the heptachlorinated bipyrrole (Q1), and brominated hexahydroxanthenes (BHDs) like TriBHD (Fig. 1).

Due to their natural origin, HNPs exhibit highly individualised distribution patterns, depending on a multitude of biogenic and abiogenic factors. These include the occurring species (including biomass and production rate), water temperature, salinity, or light availability [17–20]. Prediction of HNP contents is therefore a challenging task, as even small differences in the sampling location can result in greatly different HNP contents and patterns [21]. In contrast to many well-studied and monitored POPs, the data basis for HNPs is scarce. In the past decade (2015–2024), the number of publications on PCBs alone exceeded the number of publications on HNPs by a factor of more than 100 (number of publications for search terms “polychlorinated biphenyl*” versus “halogenated natural product*” on https://www.scopus.com between 2015 and 2024). However, ample data availability on the occurrence of substances is an important prerequisite for the identification and evaluation of potential risks.

Currently, methods analysing HNPs mostly use a combination of lipid extraction, lipid removal, and further clean-up steps (e.g., silica column chromatography), followed by analysis with gas chromatography with mass spectrometry (GC/MS) [10, 13]. Approaches combining high-performance liquid chromatography (HPLC) or GC with high-resolution MS (GC/HR-MS) were also used, especially for identifying unknown analytes [9, 22]. Often, selected HNPs are integrated into existing analytical methods for the determination of POPs [14, 23]. The lack of isotope-labelled internal standards as well as the scarce availability of analytical standards often limits the possible scope of method validation. Therefore, detailed validation data were rarely published, limiting the comparability of analytical methods and results. Especially, parameters such as repeatability and limit of quantitation (LOQ) in the matrix are necessary to enable a comprehensive evaluation of literature data (e.g., absence/presence of analytes, comparison of contents reported for different samples). Access to reliable and validated methods is an important incentive for laboratories to expand their analytical scope and incorporate HNPs into their portfolio. This can increase the knowledge about HNPs and is crucial for generating data needed for exposure and toxicological assessments.

Thus, the goal of this study was the validation of a method for analysing 17 selected HNPs in seafood, using equipment available in most laboratories that analyse organohalogen contaminants. Method validation was carried out with salmon (*Salmo salar*) and blue mussels (*Mytilus edulis*), i.e., two important matrices for HNP investigations (fish, bivalves). The validated method was then applied to a variety of seafood samples from the German retail market. In that way, both the suitability of the method for various matrices was demonstrated as well as data on the occurrence and content of HNPs were generated. This is an important first step to gain an overview of the exposure of seafood consumers to HNPs in Germany.

## Materials and methods

### Samples

Seventeen commercial seafood samples were analysed, including nine of the ten most consumed seafood products on the German market in 2024 [24]. The samples consisted of 14 different species and were purchased from German food retailers (Table S1). Method development and validation were performed with salmon fillet (*S. salar*, Norway) and blue mussel tissue (*M. edulis*, Germany). All samples were initially cut into pieces (approximately 4 cm^3^) and freeze-dried in a Lyovac GT 2 lyophilisation unit (Leybold, Cologne, Germany) for 24 h or 48 h, depending on sample amount, and then homogenised using a Grindomix GM 200 (Retsch, Haan, Germany).

### Chemicals and reagents

Standard substances used for identification and quantitation are listed in the supplementary information (Table S2). Analytes were chosen to include at least one substance of each HNP group commonly reported in marine apex predators [25]. Standard solutions were prepared in a simulated matrix using a purified commercial sunflower oil (details provided under “Quantitation and quality assurance”). Cyclohexane, ethyl acetate, *iso*-octane, dichloromethane, and *n*-hexane (all picograde) were obtained from ScienTEST-bioKEMIX (Wesel, Germany). For extraction and lipid removal by gel permeation chromatography (GPC), an azeotropic mixture (46/54, *w*/*w*) of cyclohexane and ethyl acetate was used (referred to as “C/E”) [26]. For the cleaning and deactivation of glass liners, methanol (LC–MS-grade) from Merck (Darmstadt, Germany) and dichlorodimethylsilane (99% +) from Fisher Scientific (Schwerte, Germany) were used. Sodium sulphate (> 99%) was purchased from Carl Roth (Karlsruhe, Germany) and heated to 190 °C for at least 4 h before use. Sea sand (extra pure) and silica gel 60 (0.063–0.200 mm) were ordered from Merck (Darmstadt, Germany) and heated to 450 °C and 190 °C, respectively, for at least 4 h before use. For cotton wool, a commercially available product was used, which was cleaned beforehand by soaking in C/E.

### Lipid extraction

Extraction and clean-up procedures were developed based on methods previously described by Wu et al. [8] and the final method is schematically presented in Fig. S1. For lipid extraction, freeze-dried samples were exactly weighed into a glass beaker and ground with 2 g sodium sulphate. The sample weight was chosen based on the labelled fat content to ideally obtain 400 to 800 mg of extracted fat. For low-fat samples, a maximum of 10 g dry weight (dw) was used for sample handling reasons. The sample/sodium sulphate mixture was suspended in an aliquot of C/E (approximately 10–30 mL, depending on sample amount), taken from the total volume of 150 mL C/E used for extraction. To monitor the loss of volatile analytes, especially during subsequent concentration steps, 10 pg of perdeuterated α-hexachlorocyclohexane (*α*-PDHCH) was added to the suspension.

A chromatographic glass column (30 × 3 cm) with PTFE stopcock was stuffed with a small amount of pre-cleaned cotton wool and filled with approximately 2 cm of sea sand. A glass vessel (250 mL with 3 mL appendix) was placed at the column outlet, and the outlet of the column was opened. Then, the sample/sodium sulphate suspension was transferred into the chromatographic column. The solvent was allowed to pass through the column until the meniscus reached the sample material. The outlet was closed, and the sample material was covered with an additional layer of sea sand to avoid stirring up. The remaining C/E-mixture was added to the column, and the outlet was opened. The flow was adjusted manually to approximately 1 drop per second to allow sufficient interaction between the solvent and the sample material, resulting in a total extraction time of around 45 min.

The raw extract was concentrated to approximately 5 mL using a BÜCHI SyncorePlus Analyst (Flawi, Switzerland). The appendix of the glass vessels (3 mL) was cooled at 10 °C to avoid compound losses due to evaporation. The concentrated raw extract was transferred into a volumetric 10-mL flask, and the volume was brought up to the mark with C/E.

To be able to provide results on a lipid weight (lw) basis (i.e., ng/g lw), the lipid content was determined gravimetrically by transferring 500 µL of the 10 mL sample solution into a pre-weighed glass vial. The solvent was allowed to evaporate at room temperature, and the weight was determined again.

### Lipid removal by gel permeation chromatography (GPC)

#### GPC setup

Lipids of the raw sample extract were removed using an automated GPC system (J2 Scientific, Munich, Germany). A column (LCTech, Obertaufkirchen, Germany) filled with 22 cm of S-X3 BioBeads (corresponding to 24 g, Bio-Rad Laboratories, Hercules, CA, USA) was used for fish and invertebrates (column 1, 2 cm internal diameter, i.d.). For bivalves, another column filled with 32 cm S-X3 BioBeads (50 g) was used (column 2, 2.5 cm i.d.). The columns were operated with a flow rate of 5 mL/min of C/E as the eluent. To check column performance, salmon oil spiked with 100 ng of β-hexachlorocyclohexane (*β*-HCH), hexachlorobenzene (HCB), and octachloronaphthalene (OCN) as commercially available marker substances for early, medium, and late-eluting compounds was used [27]. The eluate was subdivided into 48 (column 1) and 90 (column 2) fractions of 30 s (2.5 mL) each. An aliquot of 0.5 mL (20%) was taken from each fraction and was subsequently analysed via gas chromatography with mass spectrometry in electron capture negative ionisation mode (GC/ECNI-MS) for spiked analytes, starting from the last fraction (column 1, fraction 48; column 2, fraction 90) until no more *β*-HCH could be detected (column 1, fraction 19; column 2, fraction 43) [8]. The remaining 2.0 mL of each fraction (80%) was used for gravimetrical determination of the fat content. In a subsequent fractionation experiment, a mix with approximately 5 ng of all analysed HNPs in solvent was injected into column 1, and the eluate was collected minute-wise from 9 to 21 min (12 fractions of 5 mL). Each fraction was concentrated to 100 µL and measured by GC/ECNI-MS.

#### Final method

A collection window of the GPC eluate was set between the start of the *β*-HCH elution and the end of the OCN elution, i.e., 10.5–22.5 min on column 1 and 22.5–41.5 min on column 2. Raw sample extracts (see “Lipid extraction”) were centrifuged (10 min, 1700 g), and 8.8 mL was injected into the GPC system (flow rate of 5 mL/min C/E). The respective fractions of column 1 and 2 were collected in a 250-mL glass vessel with 0.3 mL appendix and concentrated to approximately 1 mL using a BÜCHI SyncorePlus Analyst (Flawi, Switzerland) as described above. Before the clean-up on silica, the C/E solvent was changed to *n*-hexane. For that purpose, approximately 2 mL *n*-hexane was added to the reduced fraction, and the sample was concentrated to approximately 1 mL using the SyncorePlus Analyst (see above). This procedure was repeated two more times. The final volume of around 1 mL was used for silica clean-up.

### Silica clean-up

Deactivated silica (30% water, *w*/*w*) was prepared by mixing 70 g dried silica with 30 g water. A portion of 3 g of 30% deactivated silica was transferred to a glass column (1 cm i.d.) containing 5 mL *n*-hexane. As soon as the silica had settled, the column bed was covered with a small layer of sodium sulphate. The solvent was allowed to pass through the column until the meniscus reached the column bed, and the effluent was discarded. The sample was applied to the column using a glass pipette and eluted with 70 mL of *n*-hexane in a 250-mL glass vessel with 0.3 mL appendix. The resulting eluate was concentrated first with a SyncorePlus Analyst until a volume of < 1 mL was reached, then 15 drops of *iso*-octane were added, and the sample was transferred into a 1.5-mL glass vial. The sample was concentrated to < 100 µL with a gentle stream of nitrogen at 40 °C, and 10 µL of a solution of ^13^C_12_-PCB 153 (1 ng/mL respective 1 pg/µL) was added as an internal standard for GC/ECNI-MS analysis to compensate for inter-run variability as well as the final volume. The sample was adjusted to a final volume of approximately 100 µL using manually marked glass inserts.

### Analysis via gas chromatography with mass spectrometry (GC/ECNI-MS)

Cleaned-up sample extracts and standard solutions were analysed on a 7890/5975C GC/MS system (Agilent, Waldbronn, Germany) operated in ECNI mode. Separations were performed on a DB-5MS UI column (30 m × 0.25 mm i.d. × 0.25 µm film thickness) (Agilent, Waldbronn, Germany). One microlitre of the sample solution was injected using a programmed temperature vaporizer (PTV) injector (CIS-4, Gerstel, Mülheim, Germany). The initial injector temperature of 80 °C was held for 0.05 min and then ramped at 12 °C/s until 300 °C. The temperature was held for 2 min, and then cooled at 10 °C/min to 275 °C, where it was held for the rest of the run. Helium (purity 5.0) was used as carrier gas with a flow rate of 1.2 mL/min. The initial column oven temperature of 50 °C was held for 1 min. The oven was then heated at 10 °C/min to 150 °C, then at 5 °C/min to 200 °C, followed by 10 to 240 °C/min, and finally at 5 °C/min to 300 °C, which was held for 13 min (total run time, 50 min). The temperatures of the transfer line, the ion source, and the quadrupole were set to 300 °C, 150 °C, and 150 °C, respectively. Methane (purity 4.5, Linde, Dublin, Ireland) was used as the ECNI reagent gas at a flow rate of 2 mL/min, resulting in a source pressure of 1.2 e^−4^ torr. GC/ECNI-MS quantitation was performed in selected ion monitoring (SIM) mode using the parameters shown in Table S3 (GC retention times, quantifier, and qualifier ions). For compound confirmation, the quantifier/qualifier ion ratio in the sample had to be ± 20% compared to the ratio obtained for the analytical standard analysed in the same sequence. To ensure the good condition of the inlet system, glass liners were changed on a daily basis. Glass liners were cleaned in the ultrasonic bath in a mixture of methanol:dichloromethane:n-hexane (1:1:1, *v*/*v*/*v*) for 30 min and subsequently deactivated for 24 h in a 10% solution of dichlorodimethylsilane in *iso*-octane.

### Quantitation and quality assurance

GC/ECNI-MS quantitation was performed in MassHunter Workstation V10.2 by Agilent (Waldbronn, Germany). A one-point calibration was established for each analyte using external standard solutions with a concentration of either 100 ng/mL (100 pg/µL, mixture 1) or 10 ng/mL (10 pg/µL, mixture 2 and mixture 3, lower concentration because of low compound availability). The composition of the mixtures is provided in Table S3. The internal standard (^13^C_12_-PCB 153) was adjusted to 100 ng/mL in each case. The quotient of the peak areas obtained for the analyte and the internal standard was used for calculating analyte concentrations in the respective samples.

The standard for the one-point calibration was prepared in a 35% purified sunflower oil matrix. Specifically, 100 mg of sunflower oil was applied to the silica column and eluted with 70 mL *n*-hexane. The eluate was concentrated to 100 µL (see “Silica clean-up”). This solution was termed “100% purified sunflower oil matrix”. The “100% purified sunflower oil matrix” was diluted with *iso*-octane and the respective standard stock solutions to a final share of “35% purified sunflower oil matrix” in the respective standard mixtures.

The internal standard *α*-PDHCH (10 ng) was used for quality assurance to check for potential analyte losses during sample preparation, e.g., due to evaporation. A recovery rate of > 70% for *α*-PDHCH was set as a limit. Quantitative data of the analytes were not recovery-corrected with *α*-PDHCH.

### Method validation

Linear correlation was checked by analysing standard solutions with a 35% purified sunflower oil matrix with injections of 1 µL of 0.1, 1, 10, 100, and 500 ng/mL for mixture 1 and 0.5, 1, 5, and 10 ng/mL for mixture 2 and mixture 3. The resulting data was used for setting up a linear calibration function for the quotient of the peak areas obtained for the analyte and the internal standard ^13^C_12_-PCB 153 (100 ng/mL). For Br_4_Cl_2_-dimethyl-bipyrrole (DBP) (full name provided in Table S2), linear correlation could not be checked due to limited compound availability. For this analyte, signals in the matrix samples were in the same order of magnitude as in the standard.

Limit of detection (LOD) and LOQ were calculated by manually determining the signal-to-noise (S/N) ratio, both in solvent (10 ng/mL) and in sample solutions (salmon and blue mussel), and extrapolation to a S/N ratio of three (LOD) and ten (LOQ), respectively. Exemplary tests with stepwise dilutions indicated no discriminating effect with this procedure. To compare the values between solvent standards (in ng/mL) and samples (in ng/g lw), results of the solvent standard solutions were converted to ng/g lw using a hypothetical sample lipid weight of 800 and 400 mg for salmon and blue mussel samples, respectively.

For the determination of trueness, three non-spiked and three spiked samples of both salmon and blue mussel were prepared and analysed. Analytes were spiked into the freeze-dried material and samples were incubated for 1 h at room temperature to allow interaction of the analytes with the matrix, before grinding with sodium sulphate (see “Lipid extraction”). The native content (mean of *n* = 3) was subtracted from the content determined in the spiked samples (mean of *n* = 3) before calculating the recovery rate. Spiked amounts were 14 ng for salmon (except for 2,4,6-TBA, MHC-1, and Q1: 70 ng; to account for high native contents) and 30 ng for blue mussel (except for PCBs: 300 ng; to account for high native contents). Because of limited standard availability, Br_4_Cl_2_-DBP, BC-1 (brominated compound 1; 2,2′-dimethoxy-3,3′,5,5′-tetrabromobiphenyl), Br_5_Cl-DBP, BC-11 (2′,6-dimethoxy-2,3′,4,5′-tetrabromodiphenylether), TriBHD, Br_6_-DBP, and TetraBHD were not included in the recovery test.

To assess precision, non-spiked salmon and blue mussel samples were analysed in triplicate on 2 days. Spiking of analytes was not performed to reduce the variation to sample preparation only. Intra-day precision was determined as the mean of variation coefficients from both days (mean of two times three measurements), whereas inter-day precision was determined as the variation coefficient over all replicates (*n* = 6). No precision data was determined for 2,4-dibromophenol (2,4-dBP), 2,6-dBP, tetrabromo-1-methylpyrrole (TBMP), and BC-11 in salmon, and for Br_5_Cl-DBP, BC-11, Br_6_-DBP, and TetraBHD in blue mussel, as these analytes could not be detected above their corresponding LOQ in the analysed matrices.

The stability of the cleaned-up sample solutions was investigated by repeated analysis of a salmon and blue mussel sample extract after 0, 2 (only for salmon), 4, 8, and 16 weeks. Samples were stored at −18 °C in between. The stability of analytes’ contents was checked by the Neuman trend test. Statistical calculations were performed in Microsoft Excel Professional Plus 2021; graphics were created with SigmaPlot 14.

## Results and discussion

### Selection and adjustment of sample preparation steps

#### Lipid removal by gel permeation chromatography (GPC)

**Fig. 2:**
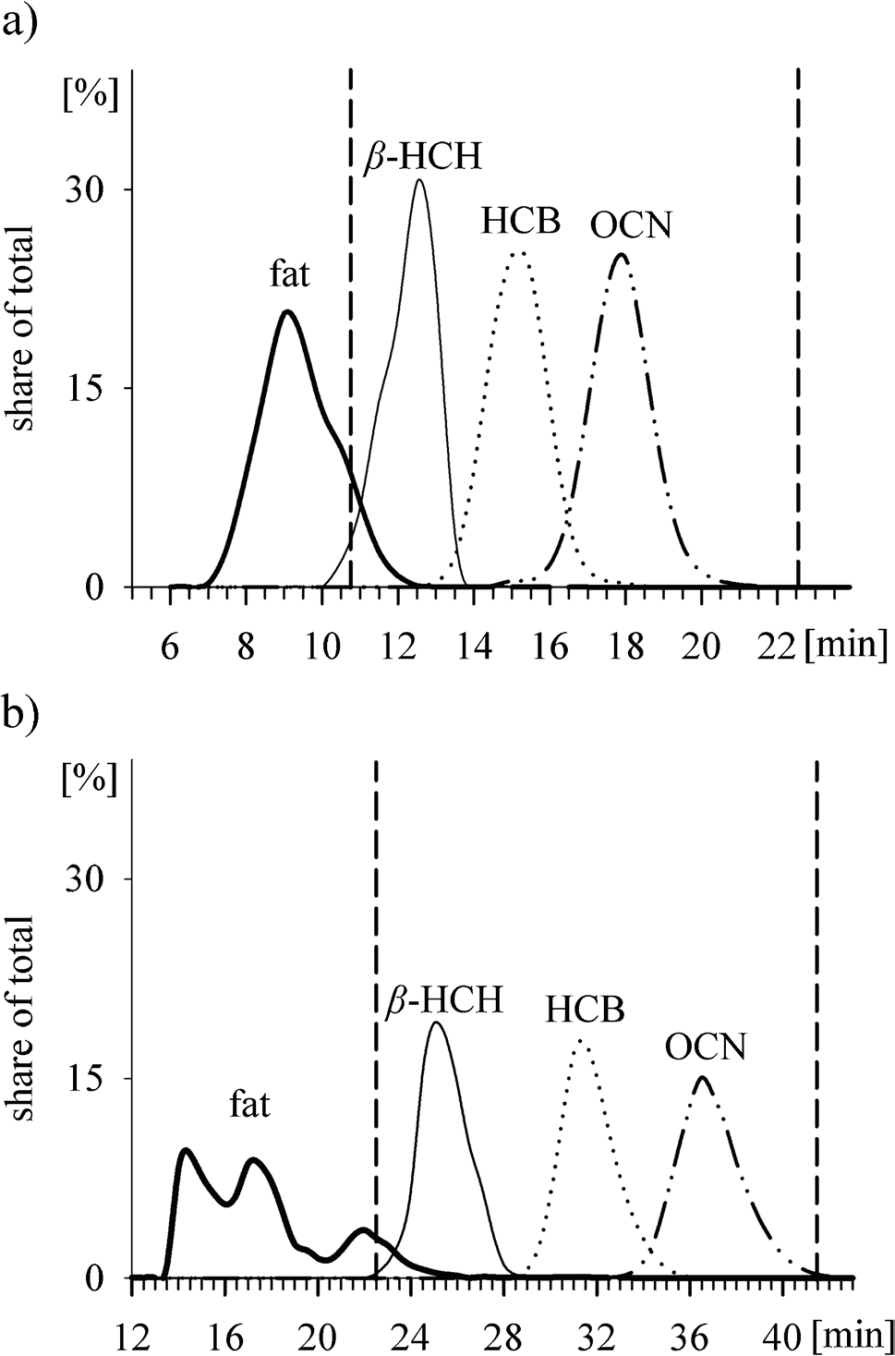
Elution of lipids and three standards of early (*β*-HCH), medium (HCB), and late (OCN) eluting compounds in **a** salmon oil on a GPC-column filled with 24 g column material (column 1) and **b** blue mussel fat on a GPC column filled with 50 g column material (column 2). Collection window (time range between dashed lines) for HNP sample preparation was set from **a** 10.5 to 22.5 min and **b** 22.5 to 41.5 min; “share of total” refers to lipid weight or peak areas for the respective analyte

Since several HNPs (e.g., BC-1, BC-2, Br_4_Cl_2_-DBP) are not stable against treatment with concentrated sulphuric acid [28], GPC was used as an alternative for lipid removal. The collection window was set using three representative POP standards (*β*-HCH, HCB, and OCN) spiked into salmon oil and blue mussel fat, as described previously [8] (further details provided in section A.1, Supplementary Information). The separation of matrix and analytes was considered sufficient with a maximum of 100 mg remaining matrix, based on a statement by Wu et al. [8]. Using column 1 (24 g BioBeads), the salmon oil matrix could be sufficiently separated without substantial loss of the POP standards (Fig. 2a). However, blue mussel lipids showed a different elution profile, and the sample matrix could not be sufficiently separated from *β*-HCH (Fig. S2). Column 2 with a higher separation capability (50 g BioBeads) was tested with this matrix and enabled a sufficient removal of mussel lipids (Fig. 2b and section A.1, Supplementary Information). Hence, the shorter column 1 (collected fraction 10.5–22.5 min) was used for fish and invertebrate samples, and the longer column 2 (collected fraction 22.5–41.5 min) for bivalves. A subsequent fractionation experiment of HNPs on column 1 confirmed that all analysed HNPs eluted within the collected fraction 10.5–22.5 min (i.e., between *β*-HCH and OCN, Table S4), demonstrating that these three POP standards indeed are suitable for assessing the GPC elution times and collection windows (Table S4).

#### Silica clean-up

Sample solutions obtained after GPC were still strongly coloured and therefore further clean-up with a silica column was performed [29]. Fractionation experiments of HNP standards with and without salmon oil showed an accelerated elution in the presence of matrix on deactivated silica (Table S5). Therefore, it was necessary to adjust the elution volume of *n*-hexane to avoid loss of the late eluting analytes, like the more polar bromophenols, for samples with low remaining fat quantities. An elution volume of 70 mL of *n*-hexane was found suitable to elute all analytes from 3 g 30% deactivated silica, even in matrix-free solution (Supplementary Information section A.2, Fig. S3).

#### Instrumental analysis

GC/MS measurement in ECNI mode was chosen because of its high sensitivity and selectivity for polyhalogenated molecules [30]. Compared to electron ionisation (EI) mode, the ECNI mode shows a reduced sensitivity and higher LODs/LOQs for lower halogenated compounds [31, 32]. However, as this work focuses on HNPs, of which most showed sufficient sensitivity, only ECNI measurements were conducted.

**Fig. 3:**
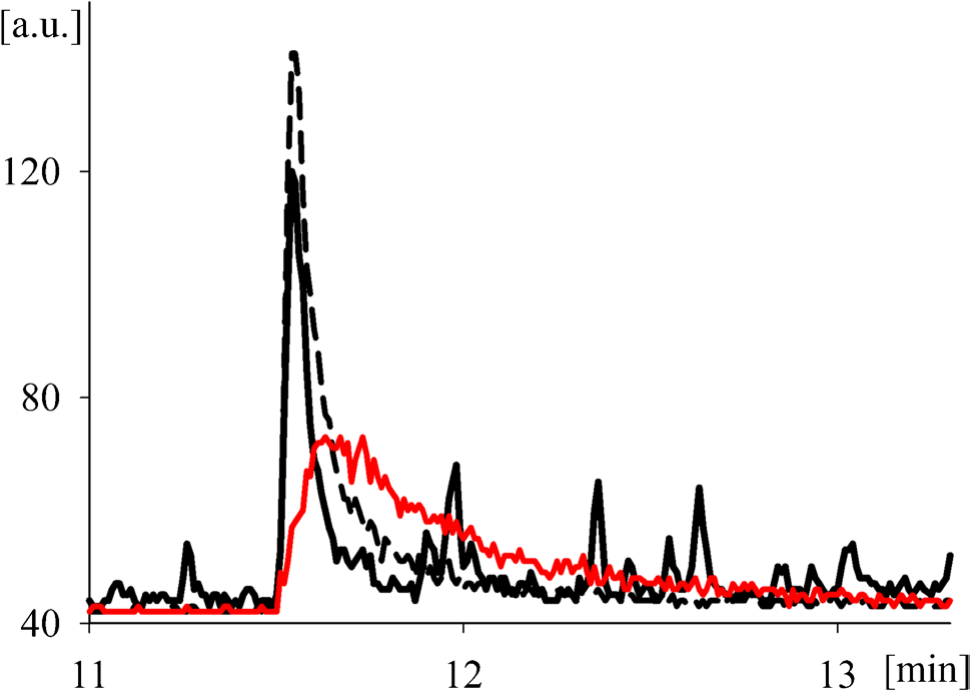
Partial GC/ECNI-MS-SIM ion chromatograms (*m/z* 79, overlay) of 2,4-dibromophenol (2,4-dBP) injected in pure *n*-hexane (red line), in 35% purified sunflower oil matrix (dashed line) (both 1 ng/mL), and its detection in an Indian squid sample (solid black line); a.u. arbitrary units

For some compound classes like bromophenols and diphenyl ethers, strong peak broadening was observed for matrix-free solvent standards (Fig. 3), as it has been previously reported [33]. However, when the standards were injected in matrix solution (here: “35% purified sunflower oil matrix”, see “Quantitation and quality assurance”), peak broadening was strongly reduced, possibly through the occupation of active sites in the liner by matrix compounds (Fig. 3).

### Method validation

#### Scope of analytes

Considering commonly reported HNPs, representatives of all substance classes except bromoindoles were included in the method (BAs, BPs, MeO-BDEs, BHDs, brominated methoxylated biphenyls (MeO-BBs) as well as polybrominated mono- and dimethyl bipyrroles (PMBP and PDBP, respectively)) [25, 34]. In addition, commonly reported POPs (PCBs, *β*-HCH, HCB, and *p,p´*-DDT) were also included. Br_5_Cl-DBP and Br_6_-DBP could not be quantified in this study since the ratios between the area of analyte and internal standard (^13^C-PCB 153) varied considerably (> 50%) for measurements over the course of 3 months. Therefore, Br_5_Cl-DBP and Br_6_-DBP were only analysed qualitatively.

#### Linear correlation

The linear correlation between the quotient of the area of the analyte and the area of the internal standard ^13^C_12_-PCB 153 and the concentration was tested over a wide concentration range covering up to three orders of magnitude. As a one-point calibration was used in routine analyses, the function was forced through the origin. The correlation was found to be linear for (up to) three orders of magnitude (correlation coefficients (*R*^2^) ≥ 0.995 for all analytes, except TetraBHD (0.968), Fig. S4–S15). For Br_5_Cl-DBP and Br_6_-DBP, no linear correlation was checked as they were not quantified.

#### Limit of detection (LOD)/limit of quantitation (LOQ)

LOD (S/N > 3) and LOQ (S/N > 10) values in solvent varied over a wide range from 0.01 to 3.33 ng/mL (Tables S6 and S7). Analytes with a high GC/ECNI-MS response could be quantified at 0.01 ng/g lw (e.g., HCB and TBMP in both salmon and blue mussel), whereas analytes with low response, such as TriBHD and TetraBHD, could be quantified only above 0.8 ng/g lw in salmon (both analytes) and 1.4 ng/g lw and 1.6 ng/g lw, respectively, in blue mussel. PCB 28 and PCB 52 showed a low GC/ECNI-MS response due to their low degree of chlorination [30]. Furthermore, these analytes form unspecific fragments of *m/z* 35 and 37 (Table S3), which are more susceptible to high background noise. Consequently, the LODs and LOQs of the two compounds in samples (0.14–1.57 ng/g lw) were among the highest values determined for all analytes investigated (Tables S6 and S7).

LOD and LOQ in matrix-free solvent standards were transformed to values in ng/g lw with the assumption of a sample fat weight of 800 mg (typical for, e.g., salmon) and 400 mg (typical for, e.g., blue mussel) (see also Annotation to Tables S6 and S7 in the Supplementary Information). This approach resulted in comparable data for solvent standards and matrix samples (Tables S6 and S7). For example, for MHC-1, the value of 0.51 ng/mL in standard solution corresponded to 0.06 and 0.11 ng/g lw for 800 mg and 400 mg sample fat weight, respectively. The corresponding extrapolated values in matrix are 0.11 and 0.14 ng/g lw for salmon and blue mussel, respectively. The results show that estimating LODs and LOQs from matrix-free standards could be a reasonable approach in case reference compounds are not available in sufficient amounts to perform spiking experiments or matrix with natural contents is not available. Furthermore, it underlines the necessity to provide the approximate fat amount used for sample preparation along with the LODs and LOQs to enable an evaluation of the respective limits when contents are given in relation to lipid weight.

#### Trueness

Three spiked samples (all analytes except Br_4_Cl_2_-DBP, BC-1, Br_5_Cl-DBP, BC-11, and Br_6_-DBP) of salmon and blue mussel resulted mostly in recovery rates between 70 and 120% (Table 1), a common quality criterion for multi-analyte methods [35]. Thus, the method can be considered suitable for the recovery of HNPs in the respective seafood matrices. Analytes with lower recovery rates were 2,6-dBP (58% in salmon/52% in blue mussel), 2,4-dBA (68%/56%), 2,4,6-TBA (65%/60%), 2,4-dBP (64%, only in salmon), and BC-3 (63% only in mussels) (Table 1). Keeping in mind the complex sample preparation including multiple evaporation steps, lower recoveries of volatile analytes were deemed acceptable. Correction of the BPs’ and BAs’ concentrations by the recovery of *α*-PDHCH resulted in an increased variation of the results. Therefore, concentrations were not corrected, but are reported as obtained. Consequently, contents of these analytes should be considered conservative.

#### Precision

Intra-day and inter-day precisions of non-spiked salmon and blue mussel samples analysed in triplicate on two different days ranged from 3 to 15% and 4 to 15%, respectively (Table 1). Values < 20% were considered appropriate for complex sample preparation protocols [35]. Due to their absence in the samples (< LOQ), no precision data could be established for TBMP and BC-11 in salmon, as well as for Br_4_Cl_2_-DBP, Br_5_Cl-DBP, BC-11, Br_6_-DBP, and TetraBHD in blue mussels. For PCB 28 and PCB 52, an unequivocal identification was hindered by their low contents in combination with unspecific fragment ions and low GC/ECNI-MS responses. Thus, these analytes were excluded from further evaluation, and no precision data are provided for these two compounds.

**Table 1:** Coefficients of variation (CV) for intra-day and inter-day precision as well as recovery rates for salmon and blue mussel

Analyte	intra-day CV for salmon in %^a^	intra-day CV for blue mussel in %^a^	inter-day CV for salmon in %^b^	inter-day CV for blue mussel in %^b^	recovery for salmon in % ± SD^c^	recovery for blue mussel in % ± SD^c^
2,4-dBP	/	8.9	/	8.2	64 ± 13	71 ± 16
2,6-dBP	/	4.9	/	10.5	58 ± 2	52 ± 6
2,4-dBA	7.2	4.2	7.0	5.1	68 ± 4	56 ± 4
2,4,6-TBA	5.9	3.7	5.8	5.4	65 ± 6	60 ± 1
2,4,6-TBP	5.4	6.6	5.9	5.9	75 ± 11	74 ± 3
HCB	7.6	4.2	7.0	7.6	82 ± 2	73 ± 3
*β*-HCH	6.6	4.0	7.4	4.4	79 ± 7	70 ± 5
TBMP	/	5.2	/	4.9	83 ± 3	83 ± 11
PCB 28	/	/	/	/	83 ± 3	85 ± 5
PCB 52	/	/	/	/	84 ± 3	87 ± 5
MHC-1	5.9	5.2	6.0	5.1	81 ± 12	101 ± 8
PCB 101	5.7	4.9	5.7	7.8	84 ± 3	91 ± 6
Q1	5.4	3.9	6.2	5.9	86 ± 4	96 ± 7
PCB 118	4.9	4.6	6.2	6.7	84 ± 3	96 ± 7
PCB 153	5.1	4.1	5.8	6.7	82 ± 3	86 ± 8
*p,p´*-DDT	8.3	9.0	9.6	15.4	86 ± 5	75 ± 6
PCB 138	7.1	5.5	6.8	7.3	93 ± 3	90 ± 7
PCB 180	5.0	5.5	6.1	8.1	85 ± 3	99 ± 7
Br_4_Cl_2_-DBP	14.7	/	14.1	/	/	/
BC-2	8.5	7.1	8.5	7.9	92 ± 6	99 ± 8
BC-1	7.6	4.3	7.6	8.1	/	/
BC-3	7.1	9.2	7.1	9.5	85 ± 9	63 ± 4
Br_5_Cl-DBP	9.5	/	8.8	/	/	/
BC-11	/	/	/	/	/	/
TriBHD	7.0	10.1	7.5	9.2	/	/
Br_6_-DBP	11.8	/	11.2	/	/	/
TetraBHD	14.8	/	15.5	/	/	/

*SD*, standard deviation

^a^Mean of variation coefficients of two triplicate analyses (*n* = 6)

^b^Variation coefficient of two triplicate analyses on two days (*n* = 6)

^c^Mean of the analysis of three fortified samples ± standard deviation (*n* = 3)

/, not determined

#### Stability of sample solutions

Repeated GC/ECNI-MS quantitation of a salmon and blue mussel sample extract after 2 (only for salmon), 4, 8, and 16 weeks stored at −18 °C (Tables S8 and S9) indicated no significant trend (*p* > 0.05, Neuman test) for any of the analytes. Therefore, sample solutions were considered stable under the chosen conditions for at least 16 weeks.

### Method application to commercial seafood

In total, 17 commercial seafood samples of 14 different species were analysed with the established and validated method. The diverse range of sample matrices of fish (with varying fat content), crustaceans, and molluscs demonstrated the method’s general applicability to a variety of seafood matrices. However, some matrices revealed low recoveries for the volatile internal standard *α*-PDHCH. For the whiteleg shrimp matrix, recoveries of less than the previously defined limit of 70% were obtained, also after repeated sample preparation and analysis (Table S10). For the rainbow trout from Denmark, *α*-PDHCH recovery rates of 61 to 79% were obtained (Table S10). Only for the most volatile compounds (2,4-dBA, 2,4,6-TBA), a similar trend for *α*-PDHCH recovery and the analyte content was observed (i.e., higher analyte contents with increasing *α*-PDHCH recovery). The difference between the highest and lowest contents was equal to or lower for the analytes (26% and 9.0%, respectively) than for the internal standard (30%), implying that no extensive analyte loss occurred compared to *α*-PDHCH. The anthropogenic compounds *β*-HCH and HCB did not follow the *α*-PDHCH trend, indicating that a loss of the internal standard does not necessarily correspond to a loss of all volatile analytes per se. Therefore, it was decided to include three samples with recoveries for *α*-PDHCH of less than 70% into the results (whiteleg shrimp, Indian squid, and oyster with 64, 47, and 65% recovery rates, respectively), although it should be considered that contents of the most volatile compounds (BPs, BAs) might be slightly underestimated in these samples.

In the following, all values larger than 1 ng/g lw are given with two significant figures (see also Tables 2 and S11). Cited values are presented as given in the corresponding literature.

#### Most common HNPs

**Fig. 4:**
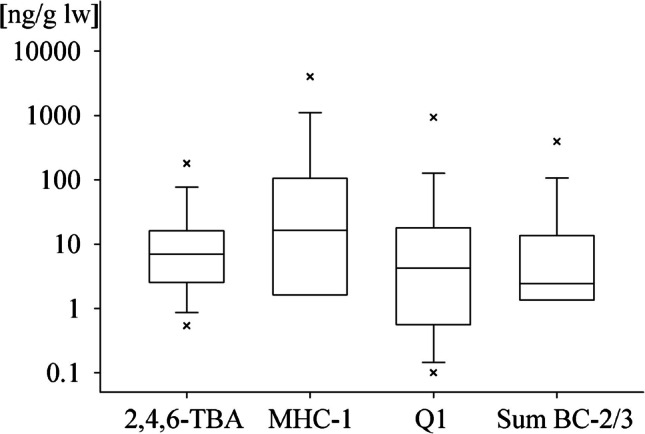
Boxplot of contents of HNPs with highest median values over all samples. Boxes range from 25 to 75th percentile with median shown. Whiskers show 10th and 90th percentile. Outliers are marked with x

HNPs were detected in all samples analysed, with contents varying widely between species and origin. Most frequently detected HNPs with the highest median values over all samples were 2,4,6-TBA, MHC-1, Q1, and BC-2/3 (Fig. 4).

Q1 and 2,4,6-TBA could be quantified in all samples (Table 2). Q1 contents ranged from less than 0.5 ng/g lw in pangasius, Venus clam, and northern prawn to more than 900 ng/g lw in oyster, with a median value of 3.4 ng/g lw (Fig. 4, Table 2). The producer of Q1 has not been identified yet, but the data obtained within this study (low contents in freshwater aquaculture products such as pangasius and Venus clam), as well as literature studies, strongly suggest a marine origin of this HNP [2, 6]. The high content in oysters from the French Atlantic Coast (900 ng/g lw, corresponding to 9.1 ng/g ww) is consistent with literature values for Q1 in blue mussels sampled from the same location and in oysters sampled approximately 270 km further to the west than the sample investigated in this study (2.4 and 11.4 ng/g ww, respectively, semi-quantified) [23]. In contrast, the sample of blue mussels from the German North Sea Coast only contained 34 ng/g lw Q1, a possible indication of high local variability of Q1 contents.

Contents of 2,4,6-TBA ranged from less than 1 ng/g lw in northern prawn and Alaska pollock to high values in Norwegian salmon and green-lipped mussel (65 and 180 ng/g lw, respectively), with a median value of 6.0 ng/g lw (Fig. 4, Table 2). High 2,4,6-TBA contents for farmed salmon and green-lipped mussel are in agreement with data from Hiebl et al*.*, who found up to 90 and 250 ng/g lw, respectively [36]. 2,4-dBA was found in all but two samples (northern prawn, Atlantic cod), which both also showed low 2,4,6-TBA contents. Samples with high 2,4,6-TBA contents often displayed elevated 2,4-dBA contents (Pearson correlation coefficient *R* = 0.63) (Table 2). The high detection frequency of bromoanisoles confirms reports about the widespread occurrence of this compound class in marine environments [2]. On the contrary, the BPs could only be quantified in crustaceans and molluscs samples. BPs are known to occur in seawater and cyanobacteria, which explains their occurrence in filter feeders like bivalves [37, 38]. Their potential for bioaccumulation, however, is limited [39]. Instead, it was demonstrated that different organisms, including fish, are able to biotransform BPs into BAs, which can explain the higher detection frequency for BAs [39, 40]. It is important to note that anthropogenic sources for 2,4,6-TBP exist (direct use as a flame retardant, UV-induced degradation of PBDEs [33]), though elevated contents in marine samples are usually attributed to natural sources [41].

MHC-1 was frequently detected, found in 14 of 17 samples. It showed the highest overall median value of 18.8 ng/g lw, with a maximum content exceeding 4000 ng/g lw in salmon from Faroe Islands aquaculture (Fig. 4, Table 2). This is, to our knowledge, the highest content reported for MHC-1 in fish to date. The packaging of the salmon from the Faroe Islands contained a second fillet, which was subsequently analysed and yielded a value of 3900 ng/g lw MHC-1, making the high value retrieved for the first fillet plausible. Because of the high content, we purchased another sample of the same product from a different lot number (harvested and frozen in August 2023 in contrast to December 2023 for the first sample set). Again, high contents of MHC-1 were found with the two fillets containing 2700 and 2800 ng/g lw. MHC-1 contents are known to be highly dependent on the presence of the primary producing macroalgae like *Plocamium cartilagineum* and can vary over relatively short distances [21, 42]. High contents for salmon from Faroese aquaculture of up to 2250 ng/g lw have previously been reported [36, 42]. Our data therefore confirm the waters around the Faroe Islands to be an MHC-1 hotspot. The second highest content of MHC-1 of the investigated sample set was 790 ng/g lw in oyster from the French Northern Coast. This corresponds to 7.6 ng/g ww, which is lower than values reported for blue mussels sampled from the same location (23 ng/g ww) or oysters from the English Channel (49 ng/g ww, both semi-quantified) [23]. For data provided on a wet weight basis, it should be taken into account, however, that shellfish samples can contain varying amounts of seawater, also depending on the handling procedures before sample preparation (e.g., removal of excess water). These variations can reduce the comparability of contents provided in different studies.

MeO-BDE were also widespread, with BC-3 being found in all but the two crustacean samples, and BC-2 being found in 14 of 17 samples. The highest values for the sum of both analytes were found in oyster, tuna, and blue mussel (400, 74, and 50 ng/g lw, respectively) (Fig. 4, Table 2). Contents of BC-3 and BC-2 showed a correlation with each other, both for all values (Pearson correlation coefficient *R* = 0.99) as well as for the data set without the aforementioned three highest contents (*R* = 0.74). This is in line with reports about their co-occurrence in primary producers like algae and sponges [40, 43, 44].

#### Fish

Salmon from marine and rainbow trout from freshwater aquaculture showed some similarities regarding their HNP profile. They contained Tri- and TetraBHD, which were not found in wild fish samples, as well as higher contents of BAs. Both species belong to the same family (*Salmonidae*) and are fed with feed containing ingredients of marine origin, such as fish meal [45]. Similar HNP profiles could therefore be an indicator for the HNP intake via commercial fish feed instead of uptake from the surrounding environment.

Pollock and Atlantic cod, both wild fish from the North-eastern Atlantic Ocean, displayed a similar HNP distribution pattern with MHC-1 being the dominant compound (90 and 160 ng/g lw, respectively) (Table 2). The other detected HNPs (BAs, TBMP, Q1, BC-3) contributed less than 10% to the load of the investigated HNPs. Compared to farmed fish, less HNPs were detected in these wild fish samples (e.g., no detection of Br_4_Cl_2_-DBP, Tri- or TetraBHD). The similar feeding behaviour [46] and their shared origin from the North-eastern Atlantic Ocean give a plausible explanation for the similar HNP profile in the investigated pollock and Atlantic cod. In contrast, Alaska pollock from the North-eastern Pacific Ocean showed a different profile with comparably low contents of MHC-1 and Q1 (7.3 and 0.6 ng/g lw, respectively), but the highest Br_4_Cl_2_-DBP content of all samples (19 ng/g lw) (Table 2).

Tuna from the Western Pacific Ocean showed some distinct features in its HNP profile. It was characterised by the absence of MHC-1 and high contents of BC-2/3 as well as BC-1. The sum of the last three analytes was 80 ng/g lw, whereas all other fish samples showed sums lower than 10 ng/g lw (Table 2). Tuna was also the only sample containing BC-11 (6.8 ng/g lw) in a similar content as previously reported for tuna from the Japanese market (median of 8 ng/g lw) [47]. The Indo-Pacific realm is a known region for the occurrence of producers of a variety of MeO-BDE (e.g., BC-2/3) and dimethoxylated BDE (diMeO-BDE, e.g., BC-11) (e.g., sponges of *Dysidea* sp.) [43, 48], providing a potential explanation for the occurrence of these HNPs in the tuna sample.

The pangasius sample showed generally low HNP contents. This could result from the freshwater aquaculture environment in combination with the diet of an omnivorous freshwater fish, with high shares of plant-based feed [49]. A predominantly plant-based diet is also reflected by the lowest PCB content (referring to PCB 101, 118, 153, 138, and 180) of all analysed samples (Table 2 and S11).

#### Molluscs and crustaceans

Indian squid exhibited generally low levels of HNPs, with most of the detected HNPs being below 2 ng/g lw, except 2,4-dBA and 2,4,6-TBA, with 2.9 and 6.0 ng/g lw, respectively. It differed therefore from data in literature about Chokka squid from South Africa, in which Q1 and 2,4,6-TBP were the dominant detected HNPs, with Q1 values exceeding 100 ng/g lw [50]. While Chokka squid samples reported by Wu et al*.* were taken close to the coast [50], with a maximum distance of less than 100 km, squid fishing predominantly occurs offshore, outside the exclusive economic zones (usually > 200 sea miles or > 370 km from the coast) [51]. The sampling location likely determines the occurrence of primary producers for HNP and consequently the HNP intake of the respective species.

The two analysed crustacean samples, whiteleg shrimp and northern prawn, showed only a small scope of HNPs, with only six of the 17 analysed HNPs being detected. Both species revealed contents of MHC-1, Q1, and (traces of) TBMP (Table 2). However, distinct differences were observed for the presence of bromophenols and -anisoles. While 2,4-dBA and 2,4,6-TBA were dominating the profile in whiteleg shrimps, northern prawn showed the second highest contents (after green-lipped mussel) for 2,4-dBP and 2,4,6-TBP, with 4.8 and 9.3 ng/g lw, respectively. This is in line with recent results in literature for wild Japanese shrimp, with 2,4,6-TBP being detected in 89% of analysed shrimp samples, respectively [52].

HNP pattern and contents varied widely in the four analysed bivalve samples, Venus clam, blue mussel, oyster, and green-lipped mussel. The profile of the Venus clam from Vietnamese aquaculture was dominated by bromoanisoles and -phenols (Table 2). Contents of these HNPs were in the same range as for the blue mussel sample from the German North Sea (4–8 ng/g lw), but almost none of the other HNPs was detected in quantifiable amounts.

Most prominent HNPs in blue mussels were MHC-1, Q1, BC-3, and BC-2 with contents of 22 to 150 ng/g lw (Table 2). MHC-1 contents in blue mussels are known to be highly dependent on the exact sampling location [21, 23], an effect that was observed for salmon samples in this study. The content of 150 ng/g lw is high for blue mussels from the German North Sea coast compared to values between 2 and 33 ng/g lw reported in literature, but much lower than for specimens collected close to the German offshore North Sea island of Heligoland (up to 1893 ng/g lw [21]), a known habitat for the MHC-1 producer *Plocamium cartilagineum* [53].

Tri- and TetraBHD were not detected in the blue mussel sample from the German North Sea coast. These HNPs, however, were found with high contents in the oyster sample from the French coast, with 630 and 170 ng/g lw, respectively. Together with Q1, MHC-1, and BC-2/3, these HNPs were the main contributors to the detected HNP profile. Contents of MHC-1, Q1, and the sum of BC-2/3 (7.5, 8.9, and 3.8 ng/g ww) were comparable to values in literature for oysters from the north-western French Atlantic coast (49, 2, and 8 ng/g ww), keeping in mind that values based on wet weight are difficult to compare [23].

The HNP profile of green-lipped mussel was clearly dominated by Tri- and TetraBHD (7000 and 4200 ng/g lw), representing the highest detected values of HNPs in all samples investigated in this study. The combined content (sumBHD) of 11,000 ng/g lw exceeded more than 14 times that of the second highest sample (oyster, sumBHD = 800 ng/g lw). It also exceeds previously reported values for sumBHD in green-lipped mussel from New Zealand aquaculture of 256 ng/g lw (quantified via nonachlor) [36] and 1570 ng/g lw [54]. For deep-sea fish as well as tuna from the Mediterranean Sea, max. sumBHD of 17,530 and 15,610 ng/g lw have been reported, respectively [55, 56], making such high values for HNPs appear at least plausible. Besides BHD, the green-lipped mussel sample revealed the highest contents of all analysed samples for 2,4,6-TBA (180 ng/g lw), 2,4,6-TBP (15 ng/g lw), and TBMP (9.9 ng/g lw), implicating the presence of potent HNP producers in the respective area. On the contrary, contents of BC-2/3, BC-1, and Q1 were generally lower (all below 1 ng/g lw), both compared to the other mollusc samples as well as the entire sample set.

#### SumHNPs and comparison to sumPOPs

The median value for the overall sum of HNPs (sumHNPs) was 100 ng/g lw (Table 2). The highest sumHNPs in this study was found in green-lipped mussel (11,000 ng/g lw) and salmon from the Faroe Islands (4100 ng/g lw), resulting from extraordinarily high concentrations of single compounds (BHDs and MHC-1, respectively). The lowest sumHNPs of less than 10 ng/g lw were found in whiteleg shrimp, in line with recently published data in literature [52], and pangasius. Low contents for the latter probably result from generally low HNP contents in the mainly plant-based diet of these omnivorous fish, which is also reflected by low POP contents reported for fish from freshwater aquaculture [57, 58].

The sum of investigated POPs (sumPOPs) was generally lower than the sumHNPs, ranging from 1.3 ng/g lw in pangasius to 375 ng/g lw in blue mussel with a median of 27 ng/g lw (Table 2). There was no correlation between sumHNPs and sumPOPs (Pearson correlation coefficient *R* = 0.02). This result emphasises the different sources for the respective substance groups. The production and distribution of HNPs depend on a multitude of factors which underlie high variability (e.g., temperature, light availability, and salinity) [17, 18]. The impact of local factors on sumHNPs is also reflected by the varying ratio of sumHNPs and sumPOPs (*R*_sumHNPs/sumPOPs_) which was between 0.6 and 798 with a median ratio of 3.3 (Table 2). With the exception of pollock, blue mussel, and Venus clam, sumHNPs was always higher than sumPOPs. The highest* R*_sumHNPs/sumPOPs_ of 798 and 111 were found in green-lipped mussel and Faroese salmon, again caused by the high individual contents of BHDs and MHC-1 in these samples, respectively. It has to be considered that in this study only a fraction of the total POP contaminants was determined. However, according to literature data, the sum of the indicator PCBs (PCB 28, 52, 101, 138, 153, 180, sometimes also including PCB 118) usually accounts for a substantial share of the total PCB load (40–60%) [59–61], while PCBs typically are the dominant POP class with shares between 50 and 90% of all POPs [62–65]. Based on these data, it is reasonable to assume that with *R*_sumHNPs/sumPOPs_ of more than 100, the amount of HNPs exceeds the content of all anthropogenic POPs present in these samples. This is in accordance with other studies, which found HNPs contents exceeding anthropogenic POPs [16, 50, 66]. Therefore, it is important to consider naturally produced compounds when assessing organohalogen substances in marine samples, as otherwise a significant portion might be overlooked.

**Table 2:** Content of selected halogenated natural products (HNPs), sum of HNPs and sum of POPs and ratio of sum of HNPs and sum of POPs in analysed samples

	2,4-dBP	2,4-dBA	2,4,6-TBA	2,4,6-TBP^a^	TBMP	MHC-1	Q1	Br_4_Cl_2_-DBP	BC-2	BC-1	BC-3	BC-11	Tri BHD	Tetra BHD	sum HNPs	sum POPs^b^	Ratio sumHNPs/sumPOPs
*Marine fish*																	
Salmon – Ireland	< LOD	2.8	13	< LOR	< LOD	60	36	2.5	2.2	0.50	6.1	< LOD	31	5.4	160	40	4.0
Salmon – Faroe	< LOD	1.3	16	< LOR	< LOD	4000	11	1.3	1.4	0.33	3.1	< LOD	3.1	1.2	4100	37	110
Salmon – Norway	< LOD	5.6	65	< LOR	< LOD	44	21	1.1	1.7	0.29	2.3	< LOD	1.5	< LOQ	140	27	5.3
Tuna	< LOD	< LOQ	2.9	< LOR	0.05	< LOD	17	3.2	47	5.5	28	6.8	< LOQ	< LOD	110	4.8	23
Alaska pollock	< LOD	0.95	0.90	< LOR	0.03	7.3	0.58	19	2.2	1.2	6.2	< LOD	< LOD	< LOD	40	40	1.0
Pollock	< LOD	0.85	1.6	< LOD	0.08	90	6.7	< LOD	< LOQ	< LOD	1.6	< LOD	< LOD	< LOD	100	170	0.6
Atlantic cod	< LOQ	< LOD	1.2	< LOR	0.06	160	1.5	< LOD	< LOD	< LOD	2.8	< LOD	< LOD	< LOD	170	51	3.3
*Freshwater fish*																	
Rainbow trout – Denmark	< LOD	3.4	15	< LOR	< LOQ	26	3.4	< LOD	0.78	0.34	1.2	< LOD	2.8	1.7	55	27	2.0
Rainbow trout – Turkey	< LOD	0.26	4.2	< LOR	0.03	19	5.1	< LOD	0.59	0.22	1.1	< LOD	12	5.1	48	9.9	4.8
Pangasius	< LOD	0.44	5.2	< LOR	0.08	< LOD	0.10	< LOD	0.70	0.27	< LOQ	< LOD	< LOD	< LOD	7.1	1.3	5.6
*Molluscs and crustaceans*																	
Indian squid	1.3	2.9	6.0	1.1	0.35	0.50	1.3	< LOD	1.5	0.62	0.61	< LOD	< LOD	< LOD	16	13	1.2
Northern prawn	4.8	< LOD	0.54	9.3	< LOQ	5.1	0.15	< LOD	< LOD	< LOD	< LOD	< LOD	< LOD	< LOD	20	17	1.2
Whiteleg shrimp	< LOQ	1.8	4.5	< LOR	0.19	2.0	0.51	< LOD	< LOD	< LOD	< LOD	< LOD	< LOD	< LOD	9.4	4.2	2.2
Venus clam	2.1	2.5	8.0	7.7	0.12	< LOD	0.21	< LOD	< LOQ	< LOQ	1.1	< LOD	< LOD	< LOD	24	37	0.7
Blue mussel	3.6	4.1	8.1	4.5	0.30	150	34	< LOD	22	0.52	28	< LOD	< LOD	< LOD	290	380	0.8
Oyster	2.9	4.5	22	6.0	3.3	790	940	0.31	230	11	170	< LOD	630	170	3100	330	9.3
Green-lipped mussel	5.7	5.5	180	15	9.9	5.1	0.77	< LOD	< LOQ	0.16	0.45	< LOD	7000	4200	11000	14	800
Median	< LOD	1.8	6.0	< LOR	0.08	19	3.4	< LOD	0.78	0.29	1.6	< LOD	< LOD	< LOD	100	27	3.3

Contents provided in ng/g lw; values larger than 1 ng/g lw are given with two significant figures

^a^Because of blank values in some of the analysed blank samples, a limit of reporting (LOR) of 1.0 ng/g lw was set

^b^Sum of HCB, *β*-HCH, *p,p′*-DDT as well as PCB 101, 118, 153, 138, and 180

## Conclusion

With a comprehensive method validation, performance criteria for an analytical method for the detection of HNPs in salmon and blue mussel could be established. The validation of the method confirmed its reliability in the analysis of important classes of HNPs in seafood. Subsequent application of the method to frequently consumed seafood products from the German retail market demonstrated its applicability to a variety of different matrices of interest. HNPs were detected in all analysed samples, including four products from freshwater aquaculture (rainbow trout, pangasius, and Venus clam). Contents varied by up to four orders of magnitude, with certain HNPs (Tri-/TetraBHD, MHC-1) reaching µg/g lw in single samples. Variations were observed not only between different species but also among samples of the same species from different origins (salmon, rainbow trout). The sum of HNPs exceeded the sum of analysed POPs in 14 out of 17 samples, indicating that HNPs make up a relevant fraction of organohalogen compounds in seafood. The widespread detection of HNPs shows the principal exposure of consumers of seafood products in Germany to these compounds. The results highlight the need for further research to systematically evaluate contents of HNPs in seafood and therefore establish a basis for the assessment of potential risks.

## Supplementary Information

Below is the link to the electronic supplementary material.

Supplementary Material 1 (PDF 1.45 MB)

## Data Availability

Data are available from the authors upon reasonable request.
