# Supplementary material for: Method validation and analysis of halogenated natural products (HNPs) in seafood samples
Source: Anal Bioanal Chem. 2025 Oct 8;417(28):6501–16. doi: 10.1007/s00216-025-06141-2 (PMC12596326; doi:10.1007/s00216-025-06141-2)
Supplement: Supplementary file 1 — Supplementary Material 1 (PDF 1.45 MB) [file 216_2025_6141_MOESM1_ESM.pdf]

Supplementary Information – Analytical and Bioanalytical Chemistry

Method validation and analysis of halogenated natural products (HNPs) in seafood samples

Marco Krämer[1/2], Walter Vetter[2], Oliver Kappenstein[1], Astrid Spielmeyer[1]

[1] German Federal Institute for Risk Assessment, Department Reference Centre for Food and Feed Analysis, National Reference Laboratory for the Monitoring of Marine Biotoxins, Max-Dohrn-Str. 8-10, 10589 Berlin, Germany

[2] Institute of Food Chemistry (170b), University of Hohenheim, Garbenstraße 28, D-70599 Stuttgart, Germany

* corresponding author: marco.kraemer@bfr.bund.de

This Supplementary Information contains 11 tables and 14 figures.

List of tables

Table S1 Overview of the samples from the retail market used in this publication

Table S2 List of standard substances used for quantitation; compounds are listed in their order of elution

Table S3 Retention times as well as quantifier and qualifier ions used for GC-MS-detection

Table S4 Elution of HNPs and three POPs in solvent from a GPC column filled with 24 g BioBeads (flow 5 mL/min cyclohexane:ethylacetate (46:54, *w*/*w*))

Table S5 Elution of bromophenols on 5 g 30% deactivated silica with and without 0.1 g salmon fat

Table S6 Limit of detection (LOD) in solvent, salmon and blue mussel matrix

Table S7 Limit of quantification (LOQ) in solvent, salmon and blue mussel matrix

Table S8 Stability data from contents of HNPs and POPs (in ng/g lw) in repeated measurement of a sample solution of salmon

Table S9 Stability data from contents of HNPs and POPs (in ng/g lw) in repeated measurement of a sample solution of blue mussel

Table S10 Content of HNPs and POPs (in ng/g lw) as well as dry matter and fat content in dry matter (in %) in analysed samples

Table S11 Contents of HNPs and POPs (in ng/g lw) in a triplicate analysis of whiteleg shrimp and Danish rainbow trout

List of figures

Fig. S1 Flowchart for the final analytical method for the determination of selected halogenated natural products (HNPs) and persistent organic pollutants (POPs)

Fig. S2 Elution of lipids and two standards of early (*β*-HCH) and late (OCN) eluting compounds in blue mussel fat on GPC-column with 24 g column material

Fig. S3 Elution with *n*-hexane of 2,4,6-TBA (green), 2,4-dBA (white), 2,6-dBP (blue, slopingly striped), 2,4,6-TBP (yellow, horizontally striped) and 2,4-dBP (black) on 3 g of 30% deactivated silica without fat

Fig. S4 Correlation of signal and concentration for **2,4-dBP** (solid line, R^2 = 0.999) and **2,4-dBA** (dotted line, R^2 = 1.000)

Fig. S5 Correlation of signal and concentration for **2,6-dBP** (solid line, R^2 = 0.999) and **2,4,6-TBP** (dotted line, R^2 = 0.999) in four concentration levels (1, 10, 100 and 500 ng/mL)

Fig. S6 Correlation of signal and concentration for **2,4,6-TBA** (solid line, R^2 = 1.000) in five concentration levels (0.1, 1, 10, 100 and 500 ng/mL)

Fig. S7 Correlation of signal and concentration for **BC-2** (solid line, R^2 = 1.000) and **BC-3** (dotted line, R^2 = 1.000) in four concentration levels (1, 10, 100 and 500 ng/mL)

Fig. S8 Correlation of signal and concentration for **HCB** (solid line, R^2 = 1.000) and ***β*-HCH** (dotted line, R^2 = 1.000) in five or four concentration levels (0.1 [not for *β*-HCH], 1, 10, 100 and 500 ng/mL)

Fig. S9 Correlation of signal and concentration for **PCB 28** (solid line, R^2 = 1.000) and **PCB 52** (dotted line, R^2 = 1.000) in three concentration levels (10, 100 and 500 ng/mL)

Fig. S10 Correlation of signal and concentration for **PCB 101** (solid line, R^2 = 1.000) and **PCB 118** (dotted line, R^2 = 1.000) in five concentration levels (0.1, 1, 10, 100 and 500 ng/mL)

Fig. S11 Correlation of signal and concentration for **PCB 138** (solid line, R^2 = 1.000) and **PCB 180** (dotted line, R^2 = 1.000) in five concentration levels (0.1, 1, 10, 100 and 500 ng/mL)

Fig. S12 Correlation of signal and concentration for **PCB 153** (R^2 = 1.000) in five concentration levels (0.1, 1, 10, 100 and 500 ng/mL)

Fig. S13 Correlation of signal and concentration for **TBMP** (solid line, R^2 = 1.000), **Q1** (dashed line, R^2 = 1.000) and **MHC-1** (dotted line, R^2 = 1.000) in five or four concentration levels (0.1 [not for MHC-1], 1, 10, 100 and 500 ng/mL)

Fig. S14 Correlation of signal and concentration for **BC-1** (solid line, R^2 = 0.998) and **BC-11** (dotted line, R^2 = 0.996) in four concentration levels (0.5, 1, 5, 10 ng/mL)

Fig. S15 Correlation of signal and concentration for **TriBHD** (solid line, R^2 = 0.995) and **TetraBHD** (dotted line, R^2 = 0.968) in four or three concentration levels (0.5 [not for TetraBHD], 1, 5, 10 ng/mL)

Table S1 Overview of the samples from the retail market used in this publication

Sample name	Species	Description	Origin
Marine fish			
Salmon – Ireland	*Salmo salar*	Filet from fish from organic aquaculture	Irish West Coast
Salmon – Faroe	*Salmo salar*	Filet from fish from conventional aquaculture	Faroe Islands
Salmon – Norway	*Salmo salar*	Filet from fish from conventional aquaculture	Norway
Tuna	*Thunnus albacares*	Filet from wild caught fish	FAO 71 (Western Pacific Ocean)
Alaska pollock	*Gadus chalcogrammus*	Filet from wild caught fish	FAO 67 (Northeastern Pacific Ocean)
Pollock	*Pollachius virens*	Filet from wild caught fish	FAO 27 (Northeastern Atlantic Ocean)
Atlantic cod	*Gadus morhua*	Filet from wild caught fish	FAO 27 (Northeastern Atlantic Ocean)
Freshwater fish			
Rainbow trout – Denmark	*Oncorhynchus mykiss*	Filet from fish from conventional aquaculture	Denmark
Rainbow trout – Turkey	*Oncorhynchus mykiss*	Filet from fish from conventional aquaculture	Turkey
Pangasius	*Pangasius hypopthalmus*	Filet from fish from conventional aquaculture	Vietnam, Mekong Delta
Molluscs and crustaceans			
Indian squid	*Uroteuthis duvaucelii*	Wild caught squid	FAO 51 (Western Indian Ocean)
Northern prawn	*Pandalus borealis*	Wild caught, whole cooked shrimp	FAO 21 (Northern Atlantic Ocean)
Whiteleg shrimp	*Litopenaeus vannamei*	Whole shrimp from conventional aquaculture	Ecuador
Venus clam	*Paratapes undulatus*	Cooked mussels from conventional aquaculture	Vietnam
Blue mussel	*Mytilus edulis*	Raw mussels from conventional aquaculture	German North Sea Coast
Oyster	*Crassostrea gigas*	Raw mussels from conventional aquaculture	West Coast of Cotenin Peninsula, France
Green-lipped mussel	*Perna canaliculus*	Cooked mussels from conventional aquaculture	New Zealand

Table S2 List of standard substances used for quantitation; compounds are listed in their order of elution

**Analyte**	**Abbreviation**	**CAS number**	**Source**	**Purity**
2,4-Dibromophenole	2,4-dBP	615-58-7	Thermo Fisher Scientific (Waltham, USA)	99%
2,6-Dibromophenole	2,6-dBP	608-33-3	Sigma Aldrich (St. Louis, USA)	> 98% (GC)
2,4-Dibromoanisole	2,4-dBA	21702-84-1	Tokyo Chemical Industry (Tokyo, Japan)	> 98% (GC)
2,4,6-Tribromoanisole	2,4,6-TBA	607-99-8	Sigma Aldrich (St. Louis, USA)	99%
2,4,6-Tribromophenole	2,4,6-TBP	118-79-6	Thermo Fisher Scientific (Waltham, USA)	<= 100%
perdeuterated α-Hexachlorocyclohexane	α-PDHCH	86194-41-4	CDN Isotopes (Pointe-Claire, Canada)	> 99%
Hexachlorobenzene	HCB	118-74-1	Sigma Aldrich (St. Louis, USA)	> 98% (GC)
β-Hexachlorocyclohexane	β-HCH	319-85-7	Sigma Aldrich (St. Louis, USA)	>=98% (GC)
Tetrabromomethylpyrrole	TBMP	56454-29-6	synthesized by Gaul et al. [1]	
Polychlorinated biphenyl 28<br>2,4,4'-Trichlorobiphenyl	PCB 28	7012-37-5	LGC (Teddington, United Kingdom)	100%
Polychlorinated biphenyl 52<br>2,2',5,5'-Tetrachlorobiphenyl	PCB 52	35693-99-3	LGC (Teddington, United Kingdom)	98.8%
Mixed halogenated compound-1 (MHC-1)<br>(1S,2S,4R,5R, 1’E)-2-Bromo-1-bromomethyl-1,4-dichloro-5-(2’-chloroethenyl)-5-methylcyclohexane	MHC-1	/	isolated from seaweed [2]	> 95% (NMR)
Polychlorinated biphenyl 101<br>2,2',4,5,5'-Pentachlorobiphenyl	PCB 101	37680-73-2	LGC (Teddington, United Kingdom)	96.9%
Heptachloro-methyl-bipyrrole<br>2,3,3’,4,4’,5,5’-Heptachloro-1’-methyl-1,2’-bipyrrole	Q1	/	synthesized by Wu et al. [3]	

Polychlorinated biphenyl 118<br>2,3',4,4',5-Pentachlorobiphenyl	PCB 118	31508-00-6	LGC (Teddington, United Kingdom)	99.2%
Polychlorinated biphenyl 153<br>2,2',4,4',5,5'-Hexachlorobiphenyl	PCB 153	35065-27-1	LGC (Teddington, United Kingdom)	98%
$^{13}C_{12}$-Polychlorinated biphenyl 153	$^{13}C_{12}$-PCB 153	185376-58-3	Cambridge Isotopes Laboratories (Tewksbury, USA)	99%
p,p'- Dichlorodiphenyltrichloroethane	*p,p´*-DDT	50-29-3	Sigma Aldrich (St. Louis, USA)	99.9%
Polychlorinated biphenyl 138<br>2,2',3,4,4',5'-Hexachlorobiphenyl	PCB 138	35065-28-2	LGC (Teddington, United Kingdom)	98.3%
Polychlorinated biphenyl 180<br>2,2',3,4,4',5,5'-Heptachlorobiphenyl	PCB 180	35065-29-3	LGC (Teddington, United Kingdom)	98.2%
Brominated compound 10<br>5,5'-Dichloro-1,1'-dimethyl-3,3',4,4'-tetrabromo-2,2'-bipyrrole	Br_4Cl_2-DBP	253798-64-0	synthesized according to Gribble et al. [4]	
Brominated compound 2<br>2'-Methoxy-2,3',4,5'-tetrabromodiphenylether (2'-MeO-BDE68)	BC-2	96920-28-4	Neochema (Bodenheim, Germany)	98%
Brominated compound 1<br>2,2'-Dimethoxy-3,3',5,5'-tetrabromobiphenyl (2,2'-diMeO-BB80)	BC-1	/	synthesized by Marsh et al. [5, 6]	
Brominated compound 3<br>6-Methoxy-2,2',4,4'-tetrabromodiphenylether (6-MeO-BDE47)	BC-3	102739-99-1	Cambridge Isotopes Laboratories (Tewksbury, USA)	> 98%
Brominated compound 11<br>2',6-Dimethoxy-2,3',4,5'-tetrabromodiphenylether (2',6-diMeO-BDE68)	BC-11	/	synthesized by Marsh et al. [5, 6]	
5'-Chloro-3,3',4,4',5-pentabromo-1,1'-dimethyl-2,2'-bipyrrole	Br_5Cl-DBP	400767-00-2	Walter Vetter, University of Hohenheim (Stuttgart, Germany)	
Tribromohexahydroxanthene<br>(2S,4aS,9aS)-2,7-dibromo-4a-bromomethyl-1,1-dimethyl-2,3,4,4a,9,9a-hexahydro-1H-xanthene	TriBHD	/	isolated from sponges by Garson et al. and Melcher et al. [7, 8]	

3,3',4,4',5,5'-Hexabromo-1,1'-dimethyl-2,2'-bipyrrole	Br_6-DBP	/	synthesized and provided by Martin et al. [9]	
Tetrabromohexahydroxanthene<br>(2S,4aS,9aS)-2,5,7-tribromo-4a-bromomethyl-l,1-dimethyl-2,3,4,4a,9,9a-hexahydro-1H-xanthene	TetraBHD	/	isolated from sponges by Garson et al. and Melcher et al. [7, 8]	

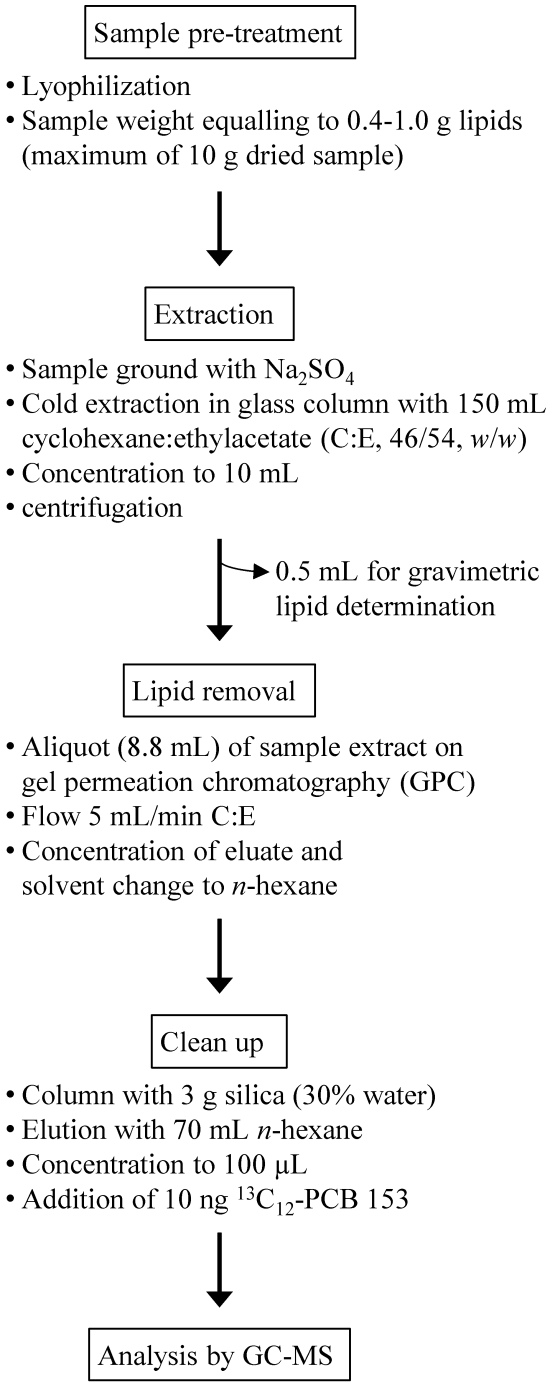

Fig. S1 Flowchart for the final analytical method for the determination of selected halogenated natural products (HNPs) and persistent organic pollutants (POPs)

Table S3 Retention times as well as quantifier and qualifier ions used for GC-MS-detection

**Analyte**	**Retention time [min]**	**Quantifier *m/z***	**Qualifier *m/z***	**Inlcuded in mixture No.**
2,4-dBP	11.55	250	79	1
2,6-dBP	11.95	250	79	1
2,4-dBA	13.32	79	81	1
2,4,6-TBA	15.75	79	81	1
2,4,6-TBP	16.25	330	332	1
α-PDHCH	17.11	261	259	1
HCB	17.26	284	250	1
β-HCH	18.15	71	35	1
TBMP	18.78	395	397	1
PCB 28	20.54	35	37	1
PCB 52	21.74	35	37	1
MHC-1	24.06	160	116	1
PCB 101	24.21	326	324	1
Q1	24.36	386	388	1
PCB 118	25.75	326	324	1
PCB 153	26.27	360	326	1
$^{13}C_{12}$-PCB 153	26.27	372	374	1/2/3
p,p´-DDT	26.93	71	35	1
PCB 138	26.94	360	326	1
PCB 180	28.69	396	360	1
Br_4Cl_2-DBP	30.11	544	546	3
BC-2	30.14	161	159	1
BC-1	30.19	79	81	2
BC-3	30.53	161	159	1
Br_5Cl-DBP	31.46	590	588	3
BC-11	31.56	79	161	2
TriBHD	32.75	79	81	2
Br_6-DBP	32.84	634	632	3
TetraBHD	36.47	544	546	2

for abbreviations see Table S2

A Method development

A.1 Lipid removal

For lipid removal, the use of GPC was adopted. The determination of the relevant fraction for HNP collection was done with three standards (100 ng each) for early (*β*-hexachlorocyclohexane, *β*-HCH), medium (hexachlorobenzene, HCB), and late (octachloronaphthalene, OCN) eluting compounds spiked on 1 g of salmon as well as blue mussel lipids, as described previously [10].

Using a standard column (24 g bio-beads, 22 cm filling height, 2 cm internal diameter), salmon lipids were detected in the fractions between 7.0 and 12.5 min, with >80% of the salmon fat eluting until 10 min. After 10 min, the early eluting compound *β*-HCH started to elute. Consequently, the remaining share of the salmon lipids overlapped with *β*-HCH (**Fig. 2a**). In fractions collected between 10.5 and 12.5 min, less than 10% (<100 mg of 1 g fat) of the initially injected lipids was detected. Since approximately 100 mg fat can be efficiently removed by column chromatography using deactivated silica [10], fractions from 10.5 min onwards were considered relevant. Starting the collection of the organohalogen fraction at 10.5 min corresponds to a slight loss of *β*-HCH (approximately 2%). Since the HNPs are expected to elute after *β*-HCH from the GPC system, this loss of *β*-HCH was considered acceptable (see also **Table 1** for recovery rates). No OCN eluted past 22 min. Consequently, the eluate from 10.5 to 22.5 min was collected for the GPC column containing 24 g material.

Mussel fat showed a different elution profile compared to salmon and the separation of fat and early eluting POPs (here: *β*-HCH) was insufficient on the standard 24 g GPC column (**Fig. S2**). Therefore, a longer column (50 g bio-beads material, 32 cm filling height, 2.5 cm internal diameter) with a higher separation capacity was used. To achieve a high analyte recovery and less than 100 mg fat in the collected organohalogen fraction, the collection window was set from 22.5 to 41.5 min (**Fig. 2b**).

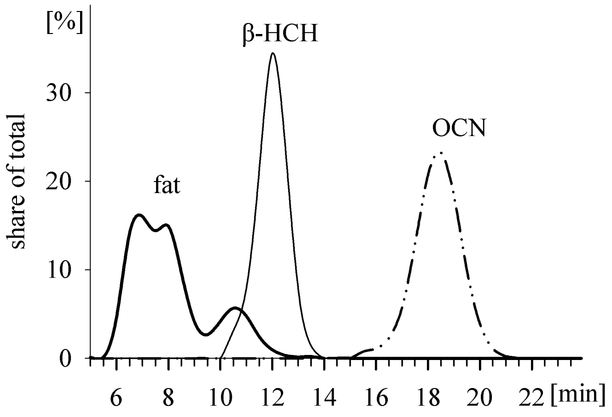

Fig. S2 Elution of lipids and two standards of early (*β*-HCH) and late (OCN) eluting compounds in blue mussel fat on GPC-column with 24 g column material

Table S4 Elution of HNPs and three POPs in solvent from a GPC column filled with 24 g BioBeads (flow 5 mL/min cyclohexane:ethylacetate (46:54, *w*/*w*))

Fraction	2,4-dBP	2,6-dBP	2,4-dBA	2,4,6-TBA	2,4,6-TBP	TBMP	MHC-1	Q1	Br_4Cl_2-DBP	BC-2	BC-1	BC-3	Br_5Cl-DBP	BC-11	Tri BHD	Br_6-DBP	Tetra BHD	*β*-HCH	HCB	OCN
9 – 10 min																				
10 – 11 min																		x		
11 – 12 min	x				x		x	x							x		x	x		
12 – 13 min	x	x			x		x	x	x	x	x	x	x	x	x		x	x		
13 – 14 min	x	x			x	x	x	x	x	x	x	x	x	x	x	x	x	x	x	
14 – 15 min	x	x	x	x	x	x	x	x	x	x	x	x	x	x	x	x	x		x	x
15 – 16 min	x	x	x	x	x	x		x	x	x	x	x	x	x	x	x	x		x	x
16 – 17 min		x	x	x	x	x				x	x	x	x	x	x	x	x		x	x
17 – 18 min			x	x		x				x	x	x							x	x
18 – 19 min				x		x														x
19 – 20 min																				x
20 – 21 min																				x

Elution range marked with x

A.2 Silica clean-up

Further clean-up of the sample extracts was performed by column chromatography using deactivated silica. For optimization, brominated anisoles and phenols were used as they were commercially available in sufficient amounts and covered the entire range of early and late eluting compounds. Of all HNPs investigated in this study, 2,4-dBP showed to highest retention. Fractionation experiments showed that the elution of the analytes was influenced by the amount of silica as well as by the presence of lipid matrix (**Table S5**, **Figure S2**). The elution was remarkably accelerated by the presence of matrix, as shown by applying the analytes in 100 mg salmon fat compared to application in solvent (**Table S5**). Depending on the lipid profile, the amount of fat remaining in the sample after GPC clean-up can vary to up to 100 mg, affecting the necessary volume of *n*-hexane for analyte elution. The final solvent volume was determined assuming a worst-case-scenario without any lipid matrix left in the sample extract after GPC. By this approach, 70 ml *n*-hexane were found suitable for analyte elution from a column of 3 g 30% deactivated silica (**Figure S2**).

Table S5 Elution of bromophenols on 5 g 30% deactivated silica with and without 0.1 g salmon fat

Fraction	Volume of *n*-hexane	2,4,6-TBA w/o fat	2,4,6-TBA with fat	2,4-dBA w/o fat	2,4-dBA with fat	2,4,6-TBP w/o fat	2,4,6-TBP with fat	2,6-dBP w/o fat	2,6-dBP with fat	2,4-dBP w/o fat	2,4-dBP with fat
1	22 mL	x	x	x	x						
2	44 mL	x				x	x	x	x		
3	66 mL					x		x			
4	88 mL									x	x
5	110 mL									x	x
6	132 mL									x	

x – compound detected by GC/MS

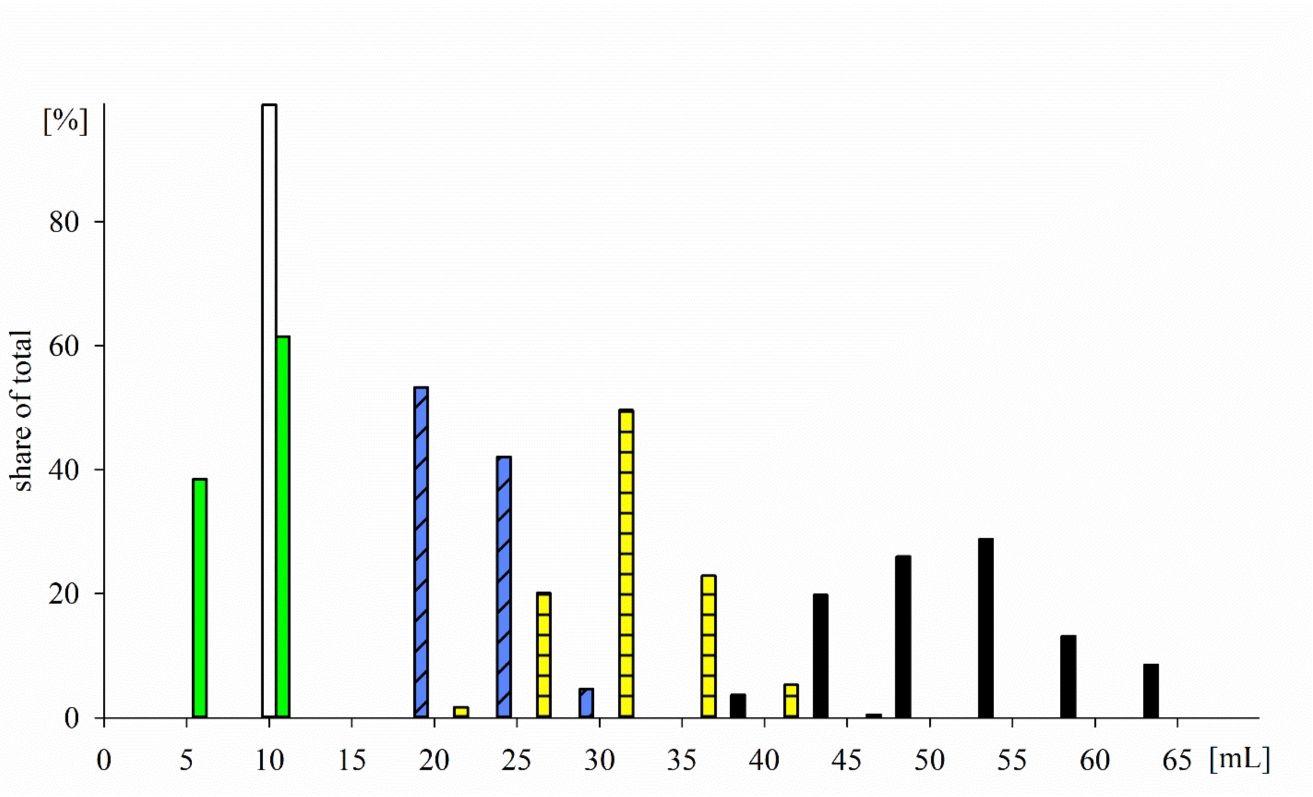

Fig. S3 Elution with *n*-hexane of 2,4,6-TBA (green), 2,4-dBA (white), 2,6-dBP (blue, slopingly striped), 2,4,6-TBP (yellow, horizontally striped) and 2,4-dBP (black) on 3 g of 30% deactivated silica without fat

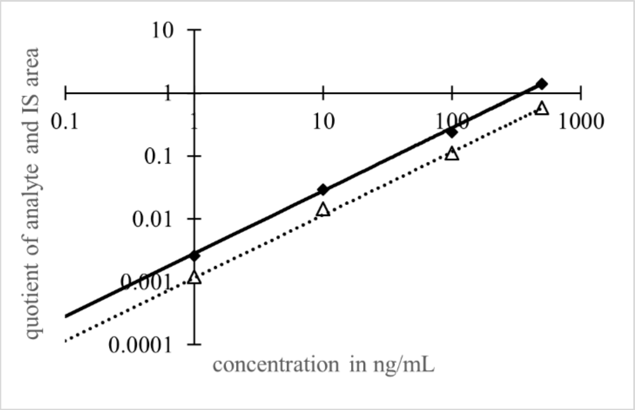

Fig. S4 Correlation of signal and concentration for **2,4-dBP** (solid line, $R^2 = 0.999$) and **2,4-dBA** (dotted line, $R^2 = 1.000$)

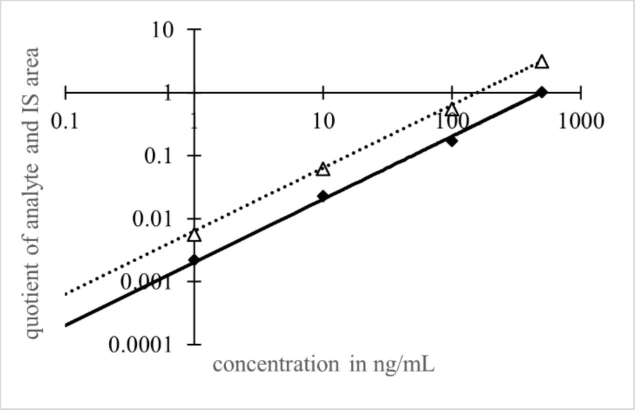

Fig. S5 Correlation of signal and concentration for **2,6-dBP** (solid line, $R^2 = 0.999$) and **2,4,6-TBP** (dotted line, $R^2 = 0.999$) in four concentration levels (1, 10, 100 and 500 ng/mL)

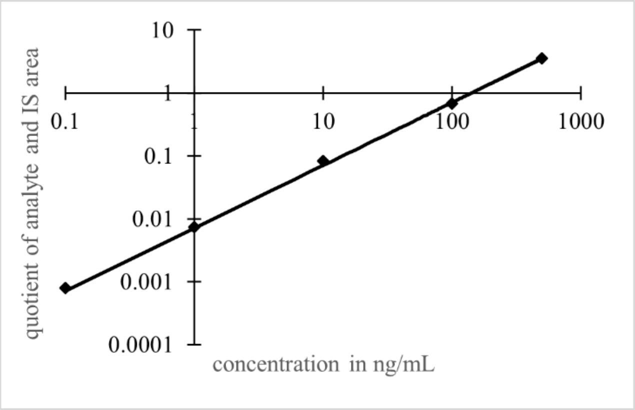

Fig. S6 Correlation of signal and concentration for **2,4,6-TBA** (solid line, $R^2 = 1.000$) in five concentration levels (0.1, 1, 10, 100 and 500 ng/mL)

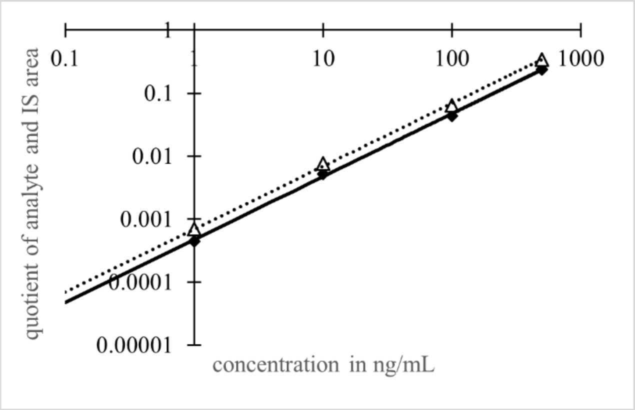

Fig. S7 Correlation of signal and concentration for **BC-2** (solid line, $R^2 = 1.000$) and **BC-3** (dotted line, $R^2 = 1.000$) in four concentration levels (1, 10, 100 and 500 ng/mL)

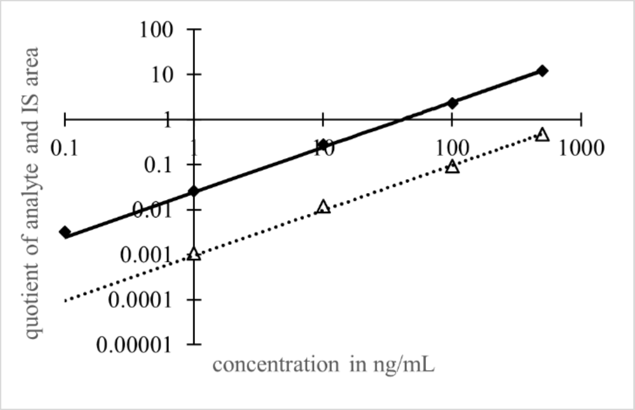

Fig. S8 Correlation of signal and concentration for **HCB** (solid line, $R^2 = 1.000$) and ***β*-HCH** (dotted line, $R^2 = 1.000$) in five or four concentration levels (0.1 [not for *β*-HCH], 1, 10, 100 and 500 ng/mL)

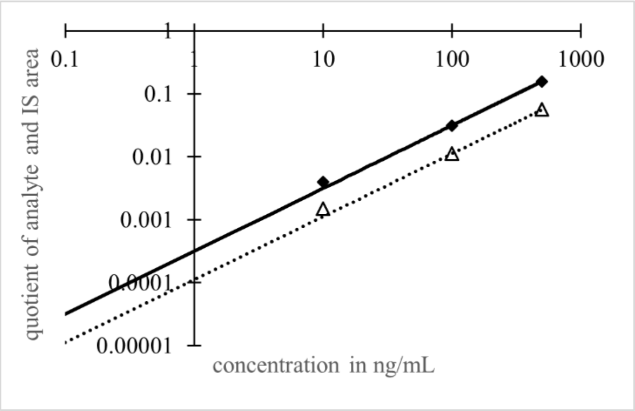

Fig. S9 Correlation of signal and concentration for **PCB 28** (solid line, $R^2 = 1.000$) and **PCB 52** (dotted line, $R^2 = 1.000$) in three concentration levels (10, 100 and 500 ng/mL)

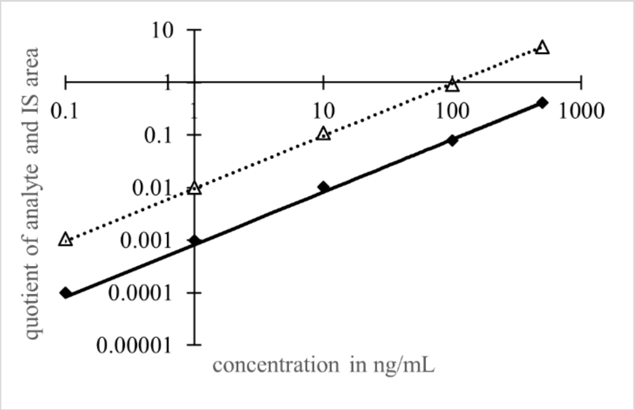

Fig. S10 Correlation of signal and concentration for **PCB 101** (solid line, R^2 = 1.000) and **PCB 118** (dotted line, R^2 = 1.000) in five concentration levels (0.1, 1, 10, 100 and 500 ng/mL)

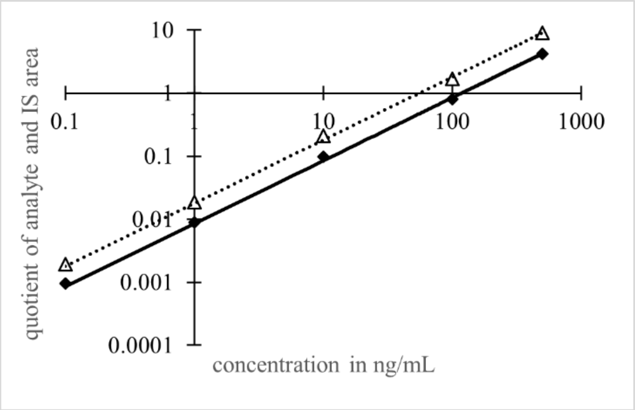

Fig. S11 Correlation of signal and concentration for **PCB 138** (solid line, R^2 = 1.000) and **PCB 180** (dotted line, R^2 = 1.000) in five concentration levels (0.1, 1, 10, 100 and 500 ng/mL)

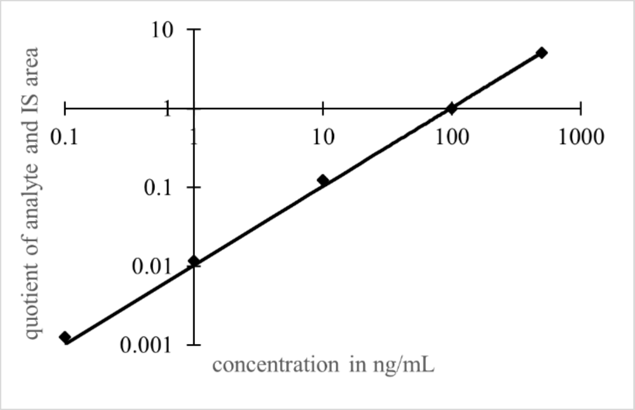

Fig. S12 Correlation of signal and concentration for **PCB 153** (R^2 = 1.000) in five concentration levels (0.1, 1, 10, 100 and 500 ng/mL)

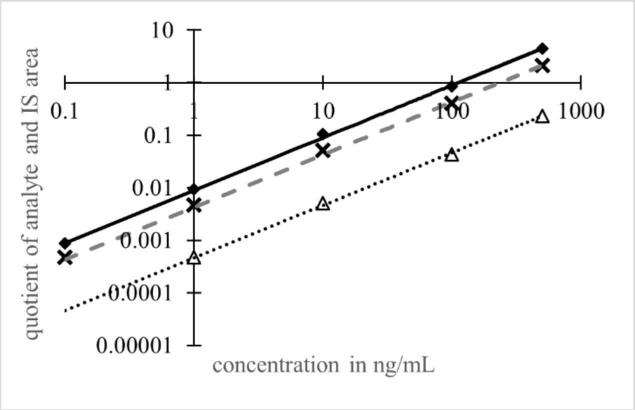

Fig. S13 Correlation of signal and concentration for **TBMP** (solid line, $R^2 = 1.000$), **Q1** (dashed line, $R^2 = 1.000$) and **MHC-1** (dotted line, $R^2 = 1.000$) in five or four concentration levels (0.1 [not for MHC-1], 1, 10, 100 and 500 ng/mL)

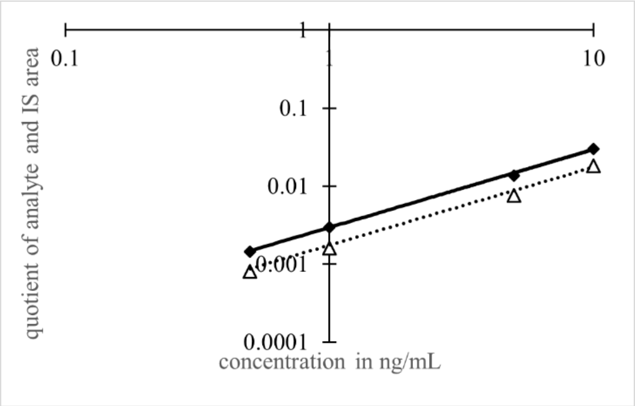

Fig. S14 Correlation of signal and concentration for **BC-1** (solid line, $R^2 = 0.998$) and **BC-11** (dotted line, $R^2 = 0.996$) in four concentration levels (0.5, 1, 5, 10 ng/mL)

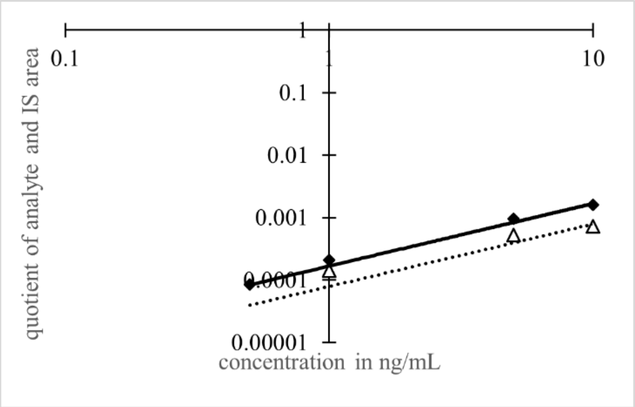

Fig. S15 Correlation of signal and concentration for **TriBHD** (solid line, $R^2 = 0.995$) and **TetraBHD** (dotted line, $R^2 = 0.968$) in four or three concentration levels (0.5 [not for TetraBHD], 1, 5, 10 ng/mL)

Annotation to **Table S6** and **Table S7**: Formula for transforming determined limit of detection (LOD) and limit of quantification (LOQ) in ng/mL solvent to values in ng/g lw in samples with a hypothetical sample lipid weight of 800 or 400 mg

$$LOD_{in\ sample} = \frac{LOD_{in\ solvent} * V}{0.88 * m}$$

$$LOQ_{in\ sample} = \frac{LOQ_{in\ solvent} * V}{0.88 * m}$$

with	$LOD/LOQ_{in\ sample}$	calculated LOD/LOQ in sample in ng/g lw
	$LOD/LOQ_{in\ solvent}$	determined LOD/LOQ (via S/N) in solvent in ng/mL
	V	volume of sample solution for analysis (0.1 mL)
	0.88	factor to account for only taking an aliquot of 8.8 mL of 10 mL for GPC
	m	sample lipid weight (either 0.4 or 0.8 g)

Table S6 Limit of detection (LOD) in solvent, salmon and blue mussel matrix

Analyte	LOD in solvent in ng/mL	LOD in solvent transformed to ng/g lw with a hypothetical fat content of 800 mg	LOD in salmon in ng/g lw with 800 mg fat content	LOD in solvent transformed to ng/g lw with a hypothetical fat content of 400 mg	LOD in blue mussel in ng/g lw with 400 mg fat content
2,4-dBP	0.21	0.03	0.02	0.06	0.05
2,6-dBP	0.25	0.04	0.01	0.07	0.03
2,4-dBA	0.12	0.02	0.03	0.03	0.07
2,4,6-TBA	0.02	0.01	0.01	0.01	0.02
2,4,6-TBP	0.03	0.01	0.02	0.01	0.01
HCB	0.01	0.01	0.01	0.01	0.01
β-HCH	0.52	0.07	0.09	0.15	0.18
TBMP	0.01	0.01	0.01	0.01	0.01
PCB 28	0.30	0.04	0.14	0.08	0.19
PCB 52	1.00	0.14	0.47	0.28	0.47
MHC-1	0.51	0.07	0.11	0.15	0.14
PCB 101	0.09	0.01	0.05	0.03	0.06
Q1	0.01	0.01	0.01	0.01	0.01
PCB 118	0.01	0.01	0.01	0.01	0.01
PCB 153	0.01	0.01	0.01	0.01	0.01
p,p´-DDT	0.33	0.05	0.12	0.09	0.11
PCB 138	0.02	0.01	0.01	0.01	0.02
PCB 180	0.01	0.01	0.01	0.01	0.01
Br_4Cl_2-DBP	0.09	0.01	0.07	0.03	0.09
BC-2	0.18	0.03	0.04	0.05	0.26
BC-1	0.01	0.01	0.04	0.01	0.04
BC-3	0.21	0.03	0.08	0.06	0.09

Br_5Cl-DBP	0.01	0.01	0.01	0.01	0.07
BC-11	0.02	0.01	0.03	0.01	0.02
TriBHD	0.04	0.01	0.23	0.01	0.47
Br_6-DBP	0.06	0.01	0.03	0.02	0.02
TetraBHD	0.58	0.08	0.24	0.16	0.41

Table S7 Limit of quantification (LOQ) in solvent, salmon and blue mussel matrix

Analyte	LOQ in solvent in ng/mL	LOQ in solvent transformed to ng/g lw with a hypothetical fat content of 800 mg	LOQ in salmon in ng/g lw with 800 mg fat content	LOQ in solvent transformed to ng/g lw with a hypothetical fat content of 400 mg	LOQ in blue mussel in ng/g lw with 400 mg fat content
2,4-dBP	0.72	0.10	0.08	0.20	0.17
2,6-dBP	0.84	0.12	0.05	0.24	0.10
2,4-dBA	0.40	0.06	0.11	0.11	0.24
2,4,6-TBA	0.06	0.01	0.03	0.02	0.08
2,4,6-TBP	0.09	0.01	0.06	0.02	0.03
HCB	0.01	0.01	0.01	0.01	0.01
β-HCH	1.72	0.24	0.30	0.49	0.60
TBMP	0.04	0.01	0.01	0.01	0.01
PCB 28	0.99	0.14	0.47	0.28	0.64
PCB 52	3.33	0.47	1.57	0.95	1.56
MHC-1	1.70	0.24	0.37	0.48	0.48
PCB 101	0.31	0.04	0.16	0.09	0.21
Q1	0.04	0.01	0.02	0.01	0.03
PCB 118	0.04	0.01	0.02	0.01	0.02
PCB 153	0.04	0.01	0.02	0.01	0.02
p,p′-DDT	1.11	0.16	0.39	0.32	0.36
PCB 138	0.07	0.01	0.03	0.02	0.06
PCB 180	0.01	0.01	0.01	0.01	0.02
Br_4Cl_2-DBP	0.30	0.04	0.23	0.09	0.31
BC-2	0.61	0.09	0.13	0.17	0.85
BC-1	0.01	0.01	0.12	0.01	0.14
BC-3	0.70	0.10	0.26	0.20	0.32
Br_5Cl-DBP	0.04	0.01	0.04	0.01	0.24
BC-11	0.05	0.01	0.09	0.02	0.05
TriBHD	0.13	0.02	0.78	0.04	1.58
Br_6-DBP	0.21	0.03	0.09	0.06	0.08
TetraBHD	1.92	0.27	0.80	0.55	1.38

Table S8 Stability data from contents of HNPs and POPs (in ng/g lw) in repeated measurement of a sample solution of salmon

Analyte	0 weeks	2 weeks	4 weeks	8 weeks	16 weeks
2,4-dBP	/	/	/	/	/
2,6-dBP	/	/	/	/	/
2,4-dBA	5.24	5.23	5.37	5.71	6.24
2,4,6-TBA	61.7	63.9	61.5	63.8	67.2
2,4,6-TBP	0.42	0.41	0.43	0.42	0.44
HCB	6.12	6.30	6.24	6.38	6.80
β-HCH	0.87	0.94	0.88	0.91	0.91
TBMP	/	/	/	/	/
PCB 28	/	/	/	/	/
PCB 52	/	/	/	/	/
MHC-1	42.4	44.6	41.4	40.5	40.5
PCB 101	2.39	2.51	2.30	2.36	2.26
Q1	20.1	21.6	19.8	20.0	19.6
PCB 118	1.81	1.96	1.80	1.81	1.83
PCB 153	5.62	5.99	5.56	5.56	5.60
p,p'-DDT	3.30	3.25	3.20	2.81	3.01
PCB 138	3.55	3.81	3.33	3.37	3.37
PCB 180	1.86	2.00	1.85	1.83	1.83
Br_4Cl_2-DBP	0.83	0.91	0.81	0.85	0.66
BC-2	0.32	0.35	0.32	0.31	0.30
BC-1	0.27	0.25	0.26	0.24	0.24
BC-3	2.18	2.40	2.13	2.15	2.11
Br_5Cl-DBP	0.02	0.02	0.02	0.02	0.02
BC-11	/	/	/	/	/
TriBHD	1.28	1.23	1.35	1.23	1.40
Br_6-DBP	0.05	0.05	0.04	0.04	0.04
TetraBHD	0.40	0.34	0.38	0.41	0.44

Table S9 Stability data from contents of HNPs and POPs (in ng/g lw) in repeated measurement of a sample solution of blue mussel

Analyte	0 weeks	4 weeks	8 weeks	16 weeks
2,4-dBP	2.03	1.82	2.31	2.05
2,6-dBP	0.28	0.28	0.33	0.29
2,4-dBA	8.52	8.12	9.26	8.37
2,4,6-TBA	31.8	29.6	33.8	31.7
2,4,6-TBP	4.36	4.05	4.36	4.51
HCB	3.45	3.41	3.68	3.71
β-HCH	17.5	16.3	18.2	16.4
TBMP	1.83	1.70	1.97	1.93
PCB 28	/	/	/	/
PCB 52	/	/	/	/
MHC-1	4.34	4.15	4.39	4.17
PCB 101	116	119	119	109
Q1	3.88	4.06	4.09	3.70
PCB 118	87.6	90.2	92.3	85.4
PCB 153	379	387	387	361
p,p´-DDT	3.45	3.38	6.73	3.02
PCB 138	212	229	225	213
PCB 180	26.6	27.7	27.7	25.9
Br_4Cl_2-DBP	/	/	/	/
BC-2	15.0	16.2	17.2	15.6
BC-1	0.48	0.49	0.44	0.41
BC-3	0.90	0.92	0.96	0.91
Br_5Cl-DBP	/	/	/	/
BC-11	/	/	/	/
TriBHD	4.52	4.07	4.69	4.46
Br_6-DBP	/	/	/	/
TetraBHD	/	/	/	/

Table S10 Contents of HNPs and POPs (in ng/g lw) in a triplicate analysis of whiteleg shrimp and Danish rainbow trout

Sample name	2,4-dBP	2,6-dBP	2,4-dBA	2,4,6-TBA	2,4,6-TBP[a]	TBMP	MHC-1	Q1	Br_4Cl_2-DBP	BC-2	BC-1	BC-3	Br_5Cl-DBP	BC-11	TriBHD	Br_6-DBP	TetraBHD
Rainbow trout – Denmark 1	< LOD	< LOD	3.4	15	< LOR	< LOQ	26	3.4	< LOD	0.78	0.34	1.2	< LOQ	< LOD	2.8	< LOD	1.7
Rainbow trout – Denmark 2	< LOD	< LOD	2.7	14	< LOR	0.01	23	3.7	< LOD	0.90	0.37	1.3	< LOQ	< LOD	2.1	< LOD	1.4
Rainbow trout – Denmark 3	< LOD	< LOD	3.2	15	< LOR	0.01	26	3.3	< LOD	1.0	0.39	1.4	< LOQ	< LOD	3.4	< LOD	1.5
Whiteleg shrimp 1	< LOQ	< LOD	1.7	4.2	< LOQ	0.19	1.9	0.50	< LOD	< LOD	< LOD	< LOD	< LOD	< LOD	< LOD	< LOD	< LOD
Whiteleg shrimp 2	< LOQ	< LOD	1.8	4.5	< LOQ	0.21	1.9	0.51	< LOD	< LOD	< LOD	< LOD	< LOD	< LOD	< LOD	< LOD	< LOD
Whiteleg shrimp 3	< LOQ	< LOD	1.8	4.5	< LOQ	0.19	2.0	0.51	< LOD	< LOD	< LOD	< LOD	< LOD	< LOD	< LOD	< LOD	< LOD

Table S10 (continued)

Sample name	HCB	*β*-HCH[b]	PCB 101	PCB 118	PCB 153	*p,p´*-DDT	PCB 138	PCB 180	Recovery of *α*-PDHCH in %
Rainbow trout – Denmark 1	3.9	1.9	3.1	2.4	7.3	1.9	4.4	1.8	79
Rainbow trout – Denmark 2	3.7	1.6	3.5	2.9	8.7	2.2	5.6	2.2	61
Rainbow trout – Denmark 3	4.5	2.5	2.9	2.3	7.3	2.5	4.8	1.8	68
Whiteleg shrimp 1	0.46	< LOD	0.90	0.29	1.1	< LOD	0.74	0.37	62
Whiteleg shrimp 2	0.49	< LOD	0.83	0.28	1.1	< LOD	0.75	0.39	59
Whiteleg shrimp 3	0.52	< LOD	0.91	0.28	1.2	< LOD	0.86	0.43	64

Values larger than 1 ng/g lw are given with two significant figures.

[a] because of blank values in some of the analysed blank samples, a limit of reporting (LOR) of 1.0 ng/g lw was set

[b] because of incomplete chromatographic separation of β-HCH and γ-HCH, amounts for β-HCH include portions of γ-HCH as well

Table S11 Content of HNPs and POPs (in ng/g lw) as well as dry matter and fat content in dry matter (in %) in analysed samples

	2,4-dBP	2,6-dBP	2,4-dBA	2,4,6-TBA	2,4,6-TBP[a]	TBMP	MHC-1	Q1	Br_4Cl_2-DBP	BC-2	BC-1	BC-3	Br_5Cl-DBP[b]	BC-11	TriBHD	Br_6-DBP[b]	TetraBHD
Marine fish																	
Salmon – Ireland	< LOD	< LOD	2.8	13	< LOR	< LOD	60	36	2.5	2.2	0.50	6.1	< LOD	< LOD	31	+	5.4
Salmon – Faroe	< LOD	< LOD	1.3	16	< LOR	< LOD	4000	11	1.3	1.4	0.33	3.1	+	< LOD	3.1	+	1.2
Salmon – Norway	< LOD	< LOD	5.6	65	< LOR	< LOD	44	21	1.1	1.7	0.29	2.3	+	< LOD	1.5	+	< LOQ
Tuna	< LOD	< LOD	< LOQ	2.9	< LOR	0.05	< LOD	17	3.2	47	5.5	27	< LOD	6.8	< LOQ	+	< LOD
Alaska pollock	< LOD	< LOD	0.95	0.90	< LOR	0.03	7.3	0.58	19	2.2	1.2	6.2	< LOD	< LOD	< LOD	< LOD	< LOD
Pollock	< LOD	< LOD	0.85	1.6	< LOD	0.08	90	6.7	< LOD	< LOQ	< LOD	1.6	< LOD	< LOD	< LOD	< LOD	< LOD
Atlantic cod	< LOQ	< LOD	< LOD	1.2	< LOR	0.06	160	1.5	< LOD	< LOD	< LOD	2.8	< LOD	< LOD	< LOD	< LOD	< LOD
Freshwater fish																	
Rainbow trout – Denmark	< LOD	< LOD	3.4	15	< LOR	< LOQ	26	3.4	< LOD	0.78	0.34	1.9	< LOD	< LOD	2.8	< LOD	1.7
Rainbow trout – Turkey	< LOD	< LOD	0.26	4.2	< LOR	0.03	19	5.1	< LOD	0.59	0.22	1.1	< LOD	< LOD	12	< LOD	5.1
Pangasius	< LOD	< LOD	0.44	5.2	< LOR	0.08	< LOD	0.10	< LOD	0.70	0.27	< LOQ	< LOD	< LOD	< LOD	< LOD	< LOD
Molluscs and crustaceans																	
Indian squid	1.3	< LOQ	2.9	6.0	1.1	0.35	0.50	1.3	< LOD	1.5	0.62	0.61	< LOD	< LOD	< LOD	< LOD	< LOD
Northern prawn	4.8	< LOD	< LOD	0.54	9.3	< LOQ	5.1	0.15	< LOD	< LOD	< LOD	< LOD	< LOD	< LOD	< LOD	< LOD	< LOD
Whiteleg shrimp	< LOQ	< LOD	1.8	4.5	< LOR	0.19	2.0	0.51	< LOD	< LOD	< LOD	< LOD	< LOD	< LOD	< LOD	< LOD	< LOD
Venus clam	2.1	2.1	2.5	8.0	7.7	0.12	< LOD	0.21	< LOD	< LOQ	0.10	1.1	< LOD	< LOD	< LOD	< LOD	< LOD
Blue mussel	3.6	< LOQ	4.1	8.1	4.5	0.30	150	34	< LOD	22	0.52	28	< LOD	< LOD	< LOD	+	< LOD
Oyster	2.9	< LOQ	4.5	22	6.0	3.3	790	940	0.31	230	11	170	+	< LOD	630	+	170
Green-lipped mussel	5.7	0.73	5.5	180	15	9.9	5.1	0.77	< LOD	< LOQ	0.16	0.45	< LOD	< LOD	7000	< LOD	4200
Median	< LOD	< LOD	1.8	6.0	< LOR	0.08	19	3.4	< LOD	0.78	0.29	1.6	< LOD	< LOD	< LOD	< LOD	< LOD

values larger than 1 ng/g lw are given with two significant figures.

[a] because of blank values in some of the analysed blank samples, a limit of reporting (LOR) of 1.0 ng/g lw was set

[b] as ratios between analyte and internal standard were found to be instable over an extended period of time, Br_6-DBP and Br_5Cl-DBP were not quantified. Instead, only qualitative results are reported.

[c] because of incomplete chromatographic separation of β-HCH and γ-HCH, amounts for β-HCH include portions of γ-HCH as well

Table S11 (continued)

Sample name	HCB	*β*-HCH[c]	PCB 101	PCB 118	PCB 153	*p,p´*-DDT	PCB 138	PCB 180	Recovery of *α*-PDHCH in %	Dry matter in %	Fat content in dry matter in %
Marine fish											
Salmon – Ireland	6.7	0.27	4.4	3.9	12	3.8	6.9	2.8	79	35.9	40.1
Salmon – Faroe	8.7	0.77	4.2	3.6	9.0	2.9	5.5	2.0	95	38.9	39.5
Salmon – Norway	6.4	0.93	2.5	1.9	5.9	3.6	3.8	2.0	76	34.6	35.3
Tuna	0.54	0.16	0.43	0.23	0.81	0.83	0.54	1.3	72	28.5	3.23
Alaska pollock	12	1.9	5.5	3.8	7.0	3.5	5.3	1.2	74	15.6	1.48
Pollock	8.2	< LOD	17	19	67	5.2	41	12	70	17.0	0.97
Atlantic cod	13	< LOD	5.8	6.2	13	2.9	8.8	2.2	78	15.3	1.18
Freshwater fish											
Rainbow trout – Denmark	3.9	1.9	3.1	2.4	7.3	1.9	4.4	1.8	79	27.2	18.5
Rainbow trout – Turkey	1.8	0.36	1.2	0.90	2.8	0.44	1.7	0.77	77	60.5	32.5
Pangasius	0.34	< LOD	0.18	0.08	0.22	0.20	0.18	0.08	70	30.7	4.83
Molluscs and crustaceans											
Indian squid	1.6	< LOD	1.4	1.4	3.3	1.4	2.6	1.6	47	10.6	2.92
Northern prawn	2.5	< LOD	2.9	2.3	5.3	< LOD	3.2	0.81	73	11.1	1.13
Whiteleg shrimp	0.52	< LOD	0.91	0.28	1.2	< LOD	0.86	0.43	64	20.2	2.35
Blue mussel	1.7	< LOD	47	35	180	6.90	97	10	74	30.2	1.97
Venus clam	14	7.0	1.0	0.43	1.3	12	0.84	0.41	82	28.9	3.34
Oyster	0.93	0.27	43	40	170	8.6	60	13	65	13.2	7.30
Green-lipped mussel	3.0	7.6	0.21	0.15	0.56	2.2	0.35	0.12	83	25.4	7.15
Median	3.0	0.36	2.9	2.3	5.9	2.9	3.8	1.6	74	27.2	3.34

References

1. Gaul S, Bendig P, Olbrich D, Rosenfelder N, Ruff P, Gaus C, Mueller JF, Vetter W. Identification of the natural product 2,3,4,5-tetrabromo-1-methylpyrrole in Pacific biota, passive samplers and seagrass from Queensland, Australia. Mar Pollut Bull. 2011;62(11):2463-8. https://doi.org/10.1016/j.marpolbul.2011.08.022
2. Vetter W, Rosenfelder N, Kraan S, Hiebl J. Structure and origin of the natural halogenated monoterpene MHC-1 and its concentrations in marine mammals and fish. Chemosphere. 2008;73(1):7-13. https://doi.org/10.1016/j.chemosphere.2008.06.020
3. Wu J, Vetter W, Gordon WG, Schneekloth JS, Blank DH, Görls H. Structure and Synthesis of the Natural Heptachloro-1'-methyl-1,2'-bipyrrole (Q1). Angew Chem Int Ed. 2002;41(10):1740-3. https://doi.org/10.1002/1521-3773(20020517)41:10<1740::AID-ANIE1740>3.0.CO;2-7
4. Gribble GW, Blank DH, Jasinsi JP. Synthesis and identification of two halogenated bipyrroles present in seabird eggs. Chem Commun. 1999:2195-6. https://doi.org/10.1039/A906655A
5. Marsh G, Stenutz R, Bergman Å. Synthesis of Hydroxylated and Methoxylated Polybrominated Diphenyl Ethers − Natural Products and Potential Polybrominated Diphenyl Ether Metabolites. European Journal of Organic Chemistry. 2003;2003(14):2566-76. https://doi.org/10.1002/ejoc.200300081
6. Marsh G, Athanasiadou M, Athanassiadis I, Bergman A, Endo T, Haraguchi K. Identification, Quantification, and Synthesis of a Novel Dimethoxylated Polybrominated Biphenyl in Marine Mammals Caught Off the Coast of Japan. Environ Sci Technol. 2005;39:8684-90. https://doi.org/10.1021/es051153v
7. Garson MJ, Manker DC, Maxwell KE, Skelton BW, White AH. Novel Bromo Metabolites From a Dictyoceratid Sponge of the Cacospongia Genus. Australian Journal of Chemistry. 1989;42(5):611-22.
8. Melcher J, Janussen D, Garson MJ, Hiebl J, Vetter W. Polybrominated hexahydroxanthene derivatives (PBHDs) and other halogenated natural products from the Mediterranean sponge Scalarispongia scalaris in marine biota. Arch Environ Contam Toxicol. 2007;52(4):512-8. https://doi.org/10.1007/s00244-006-0141-0
9. Martin R, Jäger A, Knölker H-J. Transition Metals in Organic Synthesis, Part 97: Silver-Catalyzed Synthesis of Hexahalogenated 2,2′-Bipyrroles. Synlett. 2011;2011(19):2795-8. https://doi.org/10.1055/s-0031-1289563
10. Wu Q, Schlag S, Uren R, van der Lingen CD, Bouwman H, Vetter W. Polyhalogenated Compounds (Halogenated Natural Products and POPs) in Sardine (Sardinops sagax) from the South Atlantic and Indian Oceans. J Agric Food Chem. 2020;68(22):6084-91. https://doi.org/10.1021/acs.jafc.0c01530
